# Machine learning assisted remote forestry health assessment: a comprehensive state of the art review

**DOI:** 10.3389/fpls.2023.1139232

**Published:** 2023-06-02

**Authors:** Juan Sebastián Estrada, Andrés Fuentes, Pedro Reszka, Fernando Auat Cheein

**Affiliations:** ^1^ Department of Electronic Engineering, Universidad Tecnica Federico, Santamaria, Valparaíso, Chile; ^2^ Department of Industrial Engeneering, Universidad Tecnica Federica, Santamaria, Valparaíso, Chile; ^3^ Faculty on Engineering and Science, Universidad Adolfo Ibáñez, Santiago, Chile

**Keywords:** forestry health assessment, remote sensing, machine learning, vision system, spectral information

## Abstract

Forests are suffering water stress due to climate change; in some parts of the globe, forests are being exposed to the highest temperatures historically recorded. Machine learning techniques combined with robotic platforms and artificial vision systems have been used to provide remote monitoring of the health of the forest, including moisture content, chlorophyll, and nitrogen estimation, forest canopy, and forest degradation, among others. However, artificial intelligence techniques evolve fast associated with the computational resources; data acquisition, and processing change accordingly. This article is aimed at gathering the latest developments in remote monitoring of the health of the forests, with special emphasis on the most important vegetation parameters (structural and morphological), using machine learning techniques. The analysis presented here gathered 108 articles from the last 5 years, and we conclude by showing the newest developments in AI tools that might be used in the near future.

## 1 Introduction

Climate change has increased the frequency and duration of droughts around the world (Cook et al., 2014). This has a special impact on ecosystems, where it is estimated by the United Nations Convention to Combat Desertification (UNCCD) that in the last 40 years the percentage of vegetated areas affected by droughts has doubled, and around 12 million hectares of agricultural land have been lost due to desertification (UNCCD, 2022). Another issue caused by intense droughts is the increase in wildfires. According to UNCCD (2022) more than 84% of terrestrial ecosystems are in danger due to more frequent and intensive fires. Forests are particularly affected by longer droughts due to water stress; the relationship between forestry health and posterior forest recovery is still being studied (Xu et al., 2018).

Forest management plays a fundamental role in the analysis of forest health. Its main target is to reduce risks or negative impacts derived from external disturbances (Migliavacca et al., 2021) including wildfires (Hillman et al., 2021; Reilly et al., 2021; Rodríguez et al., 2021; Wells et al., 2021; Trencanová et al., 2022), atmospheric pollution, forest stress (Cężkowski et al., 2020; Huo et al., 2021), pests (Huo et al., 2021), climate change, and forest diseases (Lin et al., 2018; Sapes et al., 2022). The scientific community has established the use of forest indicators to ease forest health assessment (Trumbore et al., 2015; Cai et al., 2021; Kopacková-Strnadová et al., 2021; Migliavacca et al., 2021; Neuville et al., 2021). These indicators comprise in their nucleus, a previous examination of factors associated with the physical and chemical forest attributes, such as greenness of the leaves, nitrogen content, tree height, canopy height, diameter at breast height, and others. Their importance lies in the study of water absorption, drought response, moisture content, changes in vegetation, and detection of tree diseases (Abdollahnejad and Panagiotidis, 2020; Raddi et al., 2021; Malabad et al., 2022; Zhuo et al., 2022).

Technological developments have allowed researchers to process massive data and obtain measurements of large portions of land. Unmanned aerial vehicles have been used in recent years as mechanisms to gather massive information about various ecosystems (Eugenio et al., 2020; Osco et al., 2021; Sangjan and Sankaran, 2021). Coupling UAVs with computer vision systems (RGB, multi-spectral, hyper-spectral and thermal cameras) and other sensors as LiDAR has allowed researchers to estimate forest parameters like height, canopy cover, DBH, vegetation indexes (Abdollahnejad and Panagiotidis, 2020; Kopacková-Strnadová et al., 2021; Raddi et al., 2021; Malabad et al., 2022; Zhuo et al., 2022). The promising use of UAVs in the assessment of forest health allows the experimentation with larger-scale satellite monitoring systems, particularly LANDSAT, SENTINEL, and even Google Earth (Ahmad et al., 2021).

Likewise, the use of remotely sensed imagery has contributed to the study of vegetation indices (Becker et al., 2018; Gallardo-Salazar et al., 2021; Rodríguez et al., 2021; Zhang Y. et al., 2021; Fakhri et al., 2022; Qiu et al., 2022; Talavera et al., 2022; Xu et al., 2022; Yang et al., 2022), forest mapping (Lin Y. Z. et al., 2021; Onishi and Ise, 2021; Fakhri et al., 2022; Li et al., 2022; Nasiri et al., 2022; Trencanová et al., 2022; Xu et al., 2022), evaluation and detection of diseased forests (Lin et al., 2018; Sapes et al., 2022), canopy characterization(Furukawa et al., 2021; Ribas Costa et al., 2022), tree species classification (Liu et al., 2021; Mäyrä et al., 2021; Onishi and Ise, 2021; Zhang C. et al., 2021; Hell et al., 2022; Yang and Kan, 2022), identification of fire-prone ecosystems (Trencanová et al., 2022), prediction of chlorophyll and nitrogen content (Yao et al., 2021; Narmilan et al., 2022; Wan et al., 2022), recognition of intrinsic forest factors (Xu et al., 2019; Dainelli et al., 2021), wildfire prevention (Trencanová et al., 2022), and so on. The analysis of these applications guarantees a comprehensive assessment of woodland features which determines the current forest health status and allows for better forest management.

In accordance with the data gathered by the different robotic platforms and sensors, it is essential to know how to treat the information. Although traditional methods such as statistical analysis are a viable option for post-processing data, currently the use of machine learning techniques has been chosen in order to generalize models, increase the accuracy of parameters estimation, and provide better feature prediction to the ecosystems variability and forest species involved (Corte et al., 2020; Wells et al., 2021; Zhang Y. et al., 2021; Ilniyaz et al., 2022; Narmilan et al., 2022; Nasiri et al., 2022; Qiu et al., 2022). In addition, some works have considered the use of deep learning strategies to further improve forest health monitoring capabilities and obtain more detailed individual tree features (Mäyrä et al., 2021; Onishi and Ise, 2021; Zhang C. et al., 2021; Hell et al., 2022; Li et al., 2022; Trencanová et al., 2022).

Machine learning (ML) models have been used as both classifiers and predictors. Forest structure parameters and tree phenotypic features are predicted using machine learning techniques with input data gathered from LiDAR, RGB, and Multi-spectral cameras (Shin et al., 2018; McClelland et al., 2019; Puliti et al., 2019; Abdollahnejad and Panagiotidis, 2020; Fan et al., 2020; Imangholiloo et al., 2020; Ahmad et al., 2021; Cai et al., 2021; Neuville et al., 2021; Sangjan and Sankaran, 2021; Yu et al., 2021). Predictions of leaf moisture, chlorophyll, and nitrogen content, have been achieved using machine learning methods (Watt et al., 2020; Lou et al., 2021; Raddi et al., 2021; Raj et al., 2021; Narmilan et al., 2022; Zhuo et al., 2022). The most common predictor is linear regression, but other common ones are support vector machine regression, random forest regression, and gradient boost machines (McClelland et al., 2019; Blanco-Sacristán et al., 2021; Fraser and Congalton, 2021b; Yu et al., 2021; Torre-Tojal et al., 2022). Another task that can be accomplished using ML methods is tree classification, which is important for forest inventory and mapping. The most common classifiers are random forests, support vector machines, and artificial neural networks (Feng et al., 2020; Guo et al., 2021; Hologa et al., 2021). Another use for classifiers in forestry health assessment is the identification of live trees and snags, the ratio between these two is an important parameter to evaluate forest health (Shovon et al., 2022).

The use of high-resolution cameras has allowed researchers to couple them with deep convolutional neural networks (Osco et al., 2021). Using deep learning structures alongside high-resolution aerial images has had good results in individual tree crown segmentation (Lin and Chuang, 2021; Onishi and Ise, 2021; Li et al., 2022). Other applications of deep convolutional neural networks are tree identification from aerial RGB and multi-spectral images, using temporal information has also been explored by researchers with the aid of recurrent convolutional neural networks (Feng et al., 2020). The most common deep learning back-bones used to perform feature extraction are, VGG19, RES-NET and Seg-Net (Pulido et al., 2020; Lin and Chuang, 2021; Hao Z. et al., 2022). Other structures used in semantic segmentation processes are U-NET and Mask-RCNN (Pulido et al., 2020).

This work presents a systematic review of scientific articles from the last five years (2017-2022) focused on forest health assessment assisted by remote sensing and machine learning techniques. For our analysis, we used Scopus (www.scopus.com) scientific database. We intend to determine which forest properties are considered to assess forest health, and how remote sensing in conjunction with machine learning strategies are used to estimate such features. Other review works related to remote sensing for forestry applications do not include information about the novel machine learning algorithms to relate the data gathered by various sensors and the expected metrics that are needed to evaluate forest health. For example, (Torres et al., 2021) describes various applications of remote sensing in the assessment of forest status and health including stress factors, plagues, tree mortality, tree decline, and tree health. However, there is no in-depth discussion about how the data is processed in those studies. A similar case is the work presented by (Guimarães et al., 2020), which covers other areas for forest management including tree classification and mapping, and tree parameter estimation; however, the processing techniques are not addressed. In Eugenio et al. (2020) it is presented a similar approach but focused on remotely piloted systems, and not considering satellite platforms that are used for the assessment of forests. A complete review of deep learning algorithms for forestry was presented in Diez et al. (2021), focusing directly on the images processing; however, such work does not present information about machine learning for regression problems. A more complete review including sensors and methods is discussed in Pérez-Cabello et al. (2021); but it is limitedto the assessment of post-fire vegetation recovery. To the best of our knowledge, our work is the only one that offers a more in-depth discussion about machine learning methods (including deep learning) and how they are implemented alongside remote sensing techniques for the assessment of forest health. **Table 1** contains a comparison between our work and previous reviews during the five-year period under study.

**Table 1:** Comparison between the present work and other similar reviews related to remote sensing in forestry applications.

Article	Years	Forest issue	Sensors	Platforms	Methods
Our Work	2017-2022	Vegetation indices, Biomass estimation, Tree structure parameters, Tree identification, Tree recognition, Water and moisture content, Chlorophyll estimation	Cameras (RGB, Hyperspectral, Multispectral, Thermal); LiDAR; TerrestrialLaser Scanning, Spectrometer	UAV, Satellite	Linear regression, Random forest, SVM, K-nearest neighbors, Deep learning
Pérez-Cabello et al. (2021)	N/A	Post-fire vegetation recovery	Cameras (RGB, Hyperspectral, Multispectral, Thermal), LiDAR, Terrestrial Laser Scanning, Spectrometer	UAV, Satellite	Not Specified
Eugenio et al. (2020)	2000-2019	Forest parameter estimation, Fire monitoring, Pest and disease detection, Natural conservation	Cameras (RGB, Hyperspectral, Multispectral, Thermal), LiDAR	UAV	Not Specified
Torres et al. (2021)	2015-2020	Forest plague detection, Forest current health, Forest health decline and mortality	Cameras (RGB, Hyperspectral, Multispectral, Thermal), LiDAR	UAV, Satellite	Random forest, SVM, K-nearest neighbors, Neural networks
(Guimarães et al., 2020)	N/A	Forest parameter estimation, Tree classification and mapping, Forest health monitoring	Cameras (RGB, Hyperspectral, Multispectral, Thermal), LiDAR	UAV	Not specified
(Diez et al., 2021)	2017-2021	Forest parameter estimation, Tree classification and mapping, Forest health monitoring	Cameras (RGB, Multispectral)	UAV	Deep learning

This paper is organized as follows: Section 2 presents the main issues and forestry problems studied using both remote sensing techniques and machine learning methods. Section 3 presents the hardware used in the assessment of forestry health, it includes both sensors and platforms. Section 4 deals with the machine learning techniques that are used to process data. Section 5 includes the discussion and the challenges that arise in the assessment of forestry health using remote sensing aided by machine learning.

## 2 Vegetative problems

**Figure 1:**
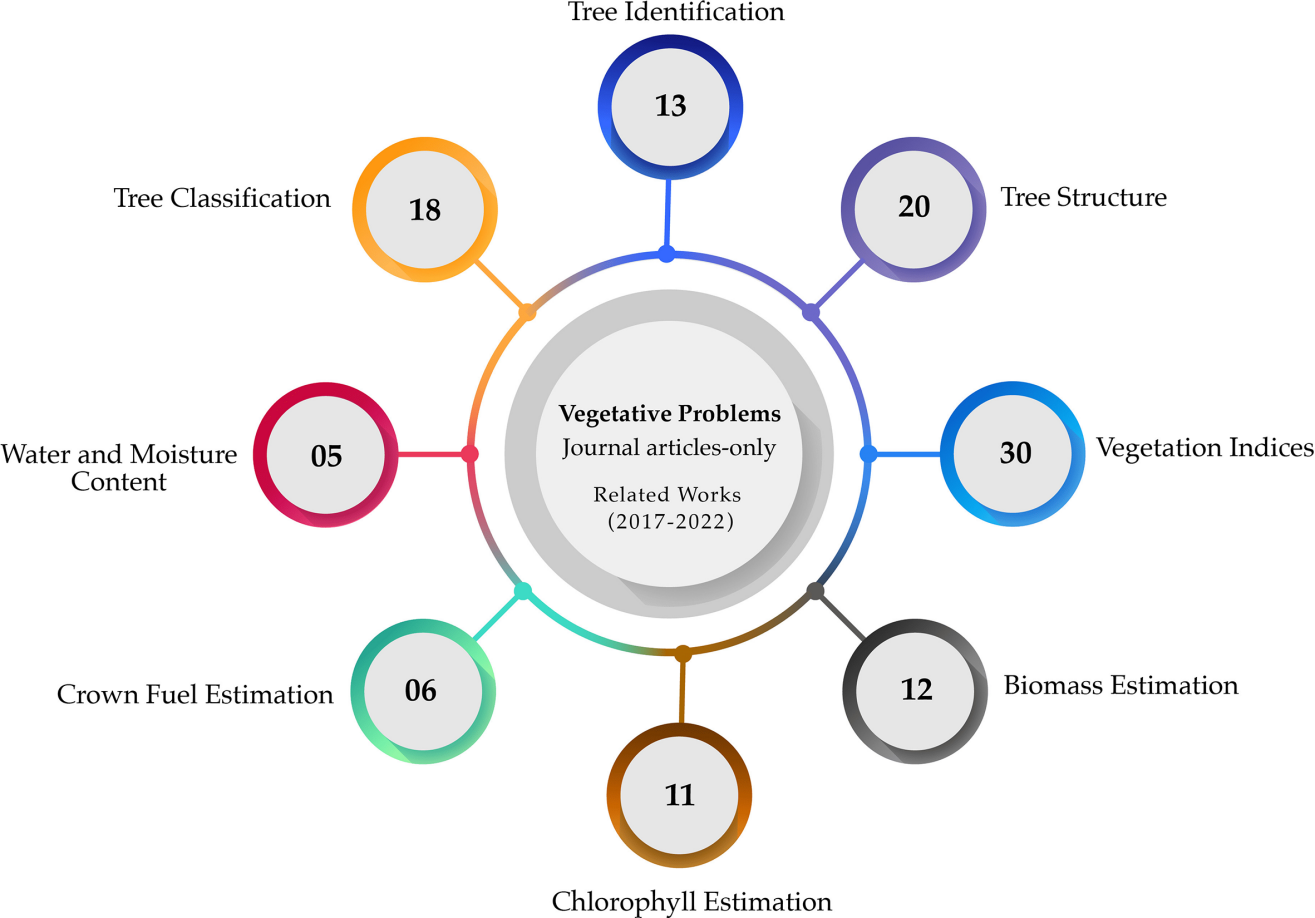
Distribution of the main vegetative problems that were studied in the reviewed articles.

This section discusses the vegetative issues that are currently being studied for forestry health assessment. In a broad sense, **Figure 1** shows the distribution of the prevalent issues that have been studied the most in the reviewed articles; these were: tree classification and identification, tree structure identification, biomass estimation chlorophyll estimation, crown fuel estimation, and water and moisture content prediction.

The first subsection is dedicated to Vegetation Indices since they are one of the most important features that help researchers predict forest and individual features from the reflected electromagnetic spectrum. The following subsection discusses tree classification and identification, tree structure parameters, biomass estimation, chlorophyll estimation, crown fuel estimation, and water and moisture content prediction.

### 2.1 Vegetation indices

A vegetation index is a mathematical transformation of two or more spectral bands that are designed to enhance a specific property or characteristic of the vegetation (Munnaf et al., 2020).

Recently, these indices have been used as input data for prediction and classification purposes alike, the spectrum of tree canopies can be considered a distinctive feature of the specific vegetation, thus making VIs useful for both vegetation identification in aerial photographs and for tree classification (Abdollahnejad and Panagiotidis, 2020; Imangholiloo et al., 2020; Yang and Kan, 2020; Guo et al., 2021; Arevalo-Ramirez et al., 2022; Cabrera-Ariza et al., 2022; Shovon et al., 2022). Photosynthetic pigments have a distinctive reflectance in some bands, thus the prediction of chlorophyll content and other pigments is suitable with the appropriate VI (Watt et al., 2020; Kopacková-Strnadová et al., 2021; Lou et al., 2021; Lu et al., 2021; Raddi et al., 2021; Raj et al., 2021; Zhuo et al., 2022). Another application using VIs is the prediction of biomass in different and (Morgan et al., 2021; Torre-Tojal et al., 2022; Yan et al., 2022).

**Tables 2A**–**D** contain the main VIs used in different studies regarding forest health, and their application; where R, G, B, NIR, and RE denote the reflectance in the Red, Green, Blue, Near Infrared, and Red Edge multi-spectral bands. Researchers focus on these five bands since most of the reviewed works use commercial infrared cameras that capture the radiation at these wavelengths. Other indices take advantage of the full spectrum and not only on specific bands but these indices are also obtained with the aid of a hyper-spectral camera or by a laboratory or hand-held spectrometer (Abdollahnejad and Panagiotidis, 2020; Watt et al., 2020; Yang and Kan, 2020; de Almeida et al., 2021; Raj et al., 2021; Villacrés and Cheein, 2022; Wan et al., 2022; Yang and Kan, 2022). Li et al. (2021) uses spectral indices to estimate the leaf water content. The authors specify five different indices: Simple Ratio, Simple Difference, normalized difference, double difference index, and difference ratio. Other indices are used to estimate the content of phosphorus and nitrogen, which is related to photosynthetic efficiency (Watt et al., 2020; Raj et al., 2021), the information gathered by hyperspectral indices, allows the processing data models to make more accurate predictions.

**Table 2A:** Common VIs used in the reviewed articles.

Vegetation Index	Formula	Application	Reference
Normalized Difference Vegetation Index (*NVDI*)	$\frac{NIR-R}{NIR+R}$	Predict forest vertical structure. Tree Recognition. Chlorophyll Content Estimation. Fuel Content Prediction.	Ahmed et al. (2021a); Raddi et al. (2021); Yu et al. (2021); Arevalo-Ramirez et al. (2022); Qiao et al. (2022); Villacrés and Cheein (2022); Zhuo et al. (2022)
Green normalized difference vegetation index (*GNDVI*)	$\frac{NIR-G}{NIR+G}$	Predict forest vertical structure. Soil Moisture Content Prediction. Chlorophyll Content estimation. Fuel Content Prediction	Yu et al. (2021); Raddi et al. (2021); Cheng et al. (2022); Arevalo-Ramirez et al. (2022); Villacrés and Cheein (2022)
Normalized difference red edge index (*NDRE*)	$\frac{NIR-RE}{NIR+RE}$	Predict forest vertical structure	Yu et al. (2021)
Structure insensitive pigment index (*SIPI*)	$\frac{NIR-B}{NIR-R}$	Predict forest vertical structure. Soil Moisture Content Prediction. Chlorophyll Content Prediction	Yu et al. (2021); Cheng et al. (2022)
Normalized green blue difference index (*NGBDI*)	$\frac{G-B}{G+B}$	Tree Classification	Guo et al. (2021)
Normalized green red difference index (*NGRDI*)	$\frac{G-R}{G+R}$	Tree Classification	Guo et al. (2021); Cabrera-Ariza et al. (2022)
Green red difference index (*GRDI*)	G−R	Tree Classification	Guo et al. (2021)
Normalized blue green vegetation index (*NBGVI*)	$\frac{B-G}{B+G}$	Tree Classification	Guo et al. (2021)
Normalized excessive green index (*NEGI*)	$\frac{2G-R-B}{2G+R+B}$	Tree Classification	Guo et al. (2021)
Modified Green Blue Vegetation Index (*MGRVI*)	$\frac{G^{2}-R^{2}}{G^{2}+R^{2}}$	Biomass Prediction	Morgan et al. (2021)
Modified Visible Atmospheric Resistant Index (*MVARI*)	$\frac{G-B}{G+R-B}$	Biomass Prediction	Morgan et al. (2021)
Red-Green-Blue Vegetation Index (*RGBVI*)	$\frac{G^{2}-B*R}{G^{2}-B*R}$	Biomass Prediction	Morgan et al. (2021)

**Table 2B:** Common VIs used in the reviewed articles.

Vegetation Index	Formula	Application	Reference
Triangular Greenness Index (*TGI*)	G−0.39R−0.61B	Biomass Prediction	Morgan et al. (2021)
Visible atmospheric resistant index (*VARI*)	$\frac{G-R}{G+R-B}$	Tree Structure. Biomass Prediction. Leaf Nitrogen Concentration	Lu et al. (2021); Morgan et al. (2021); Qiao et al. (2022)
Green red ration index (*GRRI*)	$\frac{G}{R}$	Leaf Nitrogen Concentration	Lu et al. (2021)
Normalized redness intensity (*NRI*)	$\frac{R}{R+G+B}$	Leaf Nitrogen Concentration	Lu et al. (2021)
Green Red Vegetation Index (*GRVI*)	$\frac{G-R}{G+R}$	Leaf Nitrogen Concentration. Biomass Prediction	K.C. et al. (2021); Lu et al. (2021)
Atmospherical Resistant Vegetation Index (*ARVI*)	$\frac{G-R}{G+R-B}$	Leaf Nitrogen Concentration	Lu et al. (2021)
Simple Ratio (*SR*)	$\frac{NIR}{R}$	Tree Classification. Chlorophyll Content Estimation	Abdollahnejad and Panagiotidis (2020); Zhuo et al. (2022)
Soil Adjusted Vegetation Index (*SAVI*)	$1.5\frac{NIR-R}{NIR+R+0.5}$	Tree Classification.Soil Moisture Content Prediction	Abdollahnejad and Panagiotidis (2020); Cheng et al. (2022)
Chlorophyll index (*CI*)	$\frac{NIR}{RE}-1$	Tree Classification	Abdollahnejad and Panagiotidis (2020)
Plant Sense Reflectance Index (*PSRI*)	$\frac{R-G}{RE}$	Tree Classification	Abdollahnejad and Panagiotidis (2020)
Modified canopy chlorophyll content index (*M*3*CL*)	$\frac{NIR+R+RE}{NIR-RED+RE}$	Tree Classification	Abdollahnejad and Panagiotidis (2020)
Shadow Index (*SI*)	$\frac{R+G+B}{3}$	Biomass Prediction	K.C. et al. (2021)
Modified Simple Ratio Index (*MSR*)	$\frac{NIR/R-1}{{\left(NIR/R+1\right)}^{\frac{1}{2}}}$	Soil Moisture Content Prediction	Cheng et al. (2022)
Optimized Soil Adjusted Vegetation Index (*OSAVI*)	$\frac{1.16(NIR-R)}{NIR+R+0.16}$	Soil Moisture Content Prediction. Forest Structure	Arevalo-Ramirez et al. (2022); Cheng et al. (2022)
Ratio Vegetation Index (*RVI*)	$\frac{NIR}{R}$	Soil Moisture Content Prediction	Cheng et al. (2022)
Ratio Vegetation Index 2 (*RVI*_2_)	$\frac{NIR}{G}$	Soil Moisture Content Prediction	Cheng et al. (2022)

**Table 2C:** Common VIs used in the reviewed articles.

Vegetation Index	Formula	Application	Reference
Triangular Vegetation Index (*TVI*)	60(NIR−G)−100(G−R)	Soil Moisture Content Prediction	Cheng et al. (2022)
Enhanced Vegetation Index (*EVI*)	$2.5\frac{NIR-R}{NIR+6R-7.5B+1}$	Soil Moisture Content Prediction. Forest Structure	Arevalo-Ramirez et al. (2022); Cheng et al. (2022)
Green Index (*GI*)	$\frac{G}{R}$	Soil Moisture Content Prediction	Cheng et al. (2022)
Transformed Chlorophyll Absorption in reflectance Index (*TCARI*)	$3[(RE-R)-0.2(RE-G)\frac{RE}{R}]$	Soil Moisture Content Prediction	Cheng et al. (2022)
Simple Ratio Pigment Index (*SRPI*)	$\frac{B}{R}$	Soil Moisture Content Prediction	Cheng et al. (2022)
Normalized Pigment Chlorophyll Index (*NPCI*)	$\frac{R-B}{R+B}$	Soil Moisture Content Prediction. Chlorophyll Content Estimation	Cheng et al. (2022); Zhuo et al. (2022)
Normalized Difference Vegetation Index 2 (*NDVI_GB_*)	$\frac{G-B}{G+B}$	Soil Moisture Content Prediction	Cheng et al. (2022)
Plant Senescence reflectance Index 2 (*PSRI*)	$\frac{B-R}{G}$	Soil Moisture Content Prediction	Cheng et al. (2022)
Color Index of vegetation extraction (*CIVE*)	0.44R−0.81G+0.39B+18.79	Soil Moisture Content Prediction	Cheng et al. (2022)
Near Infrared Reflectance of Vegetation (*NIR_V_*)	NIR*NDVI	Chlorophyll Content Estimation	Raddi et al. (2021)
Difference Vegetation Index (*DVI*)	NIR−R	Fuel Estimation	Villacrés and Cheein (2022)
Modified Soil Adjusted Vegetation Index (*MSAVI*)	$[2NIR+1-\sqrt{2NIR+1}-8(NIR-R)]/2$	Forest Structure	Arevalo-Ramirez et al. (2022)
Chlorophyll Absorption Reflectance Index (*CARI*)	RE−R−0.2(RE−G)	Forest Structure	Arevalo-Ramirez et al. (2022)

**Table 2D:** Common VIs used in the reviewed articles.

Vegetation Index	Formula	Application	Reference
Red Edge Modified Simple Ratio (*REMSR*)	$\frac{NIR/RE-1}{\sqrt{NIR}/RE+1}$	Forest Structure	Arevalo-Ramirez et al. (2022)
Red Edge Normalized Difference Vegetation Index (*RENDVI*)	$\frac{NIR-RE}{NIR+RE}$	Forest Structure	Arevalo-Ramirez et al. (2022)
Leaf Chlorophyll Index (*LCI*)	$\frac{NIR-RE}{NIR+R}$	Fuel Estimation	Villacrés and Cheein (2022)
Normalized Difference Red Edge (*NDRE*)	$\frac{NIR-RE}{NIR+RE}$	Fuel Estimation	Villacrés and Cheein (2022)
Red Edge Modified Simple Ratio (*REMSR*)	$\frac{NIR/RE-1}{\sqrt{NIR}/RE+1}$	Forest Structure	Arevalo-Ramirez et al. (2022)
Red Edge Normalized Difference Vegetation Index (*RENDVI*)	$\frac{NIR-RE}{NIR+RE}$	Forest Structure	Arevalo-Ramirez et al. (2022)
Leaf Chlorophyll Index (*LCI*)	$\frac{NIR-RE}{NIR+R}$	Fuel Estimation	Villacrés and Cheein (2022)
Normalized Difference Red Edge (*NDRE*)	$\frac{NIR-RE}{NIR+RE}$	Fuel Estimation	Villacrés and Cheein (2022)

Comparisons between hyper-spectral information and multi-spectral indices have been performed to evaluate drought responses in various ecosystems (Raddi et al., 2021). Other studies show that there is the possibility to recreate indices from hyper-spectral bands with the information gathered from multi-spectral indices (Villacrés and Cheein, 2022).

This section includes only a few of the most common VIs, however, more extensive articles and reviews are available, and the reader is encouraged to see (Tran et al., 2022).

### 2.2 Biomass estimation

From an ecological standpoint, biomass is defined as the mass of living organisms in a determined area or ecosystem. Biomass depending on the environment has multiple functions, for example, to know about carbon sinks and it is important in water exchange with the atmosphere. However, ecosystems are constantly changing due to climate change has strengthened environmental stressors for various ecosystems, changing the natural composition of biomass; thus estimating its value is a strong indicator of how an ecosystem responds to external changes. Biomass is also an indicator of biological fuel present in environments (Morgan et al., 2021).

### 2.3 Chlorophyll estimation

Chlorophyll concentration (CC) indicates the physiological and structural basis by which leaves drive photosynthesis (Narmilan et al., 2022) and its relationship to soil respiration (Yao et al., 2021). Likewise, studies evidence a strong connection with nitrogen content. As a matter of fact, a deficiency in nitrogen content implies a reduction in CC which improves leaf transmittance at visible wavelengths. Several findings have demonstrated that this pigment has diverse spectrum behavior with particular absorption properties at different wavelengths, thus the electromagnetic leaf reflection is an indicator of chlorophyll content. CC can be altered by natural or man-made noxious agents as well as stress factors. Additionally, an accurate measurement of CC involves a good examination of plant health, regulation of fertilizer application, and so on. CC ground measurements are used as an indicator of fertilizer status (Narmilan et al., 2022). Due to its importance in the agriculture field, current remote sensing efforts contemplate the blending of vegetation indices and machine learning techniques in order to find a well-established model that accurately defines CC (Yao et al., 2021; Narmilan et al., 2022).

### 2.4 Water and moisture content

Water and moisture content (WMC) is affected by tree species type (Yao et al., 2021) and canopy cover attributes (Gale et al., 2021). It is also a factor of soil respiration. In addition, WMC is associated with the production of CO_2_ in soil and the transportation of CO_2_ from soil to the atmosphere, so continuous or unexpected changes in WMC can affect soil respiration behaviors (Yao et al., 2021). Likewise, WMC is commonly used to assess wildfire risk in forested areas, (Barmpoutis et al., 2020; Gale et al., 2021) and knowledge of its behavior are necessary for land management decision-making (Barber et al., 2021).

Parameters such as moisture of forest canopy are used jointly with the moisture of the soil-litter layer and forest temperature for the early detection of forest fires. Therefore the development and usage of aerial remote sensing platforms including radiometer sensors, which is useful for determining and classifying areas of forests that are prone to wildfires (Varotsos et al., 2020).

The WMC is highly dependent on temperature changes, so predictive models to estimate WMC are altered by meteorological conditions (Gale et al., 2021). Current efforts are mainly focused on establishing more accurate and affordable measure systems; the most remarkable developments which have enabled effective estimation of WMC are related to the improvement of processing software/techniques and computational power and the availability of aerial imagery from satellite data, airplanes, or unmanned aerial vehicles (UAVs) (Forbes et al., 2022). Furthermore, recent studies have shown that reflectance data in a variety of wavelengths is a promising alternative for WMC estimation (Barber et al., 2021).

### 2.5 Tree recognition

The tree identification problem is to identify each individual tree from an aerial image. Its importance relies on the fact that tree recognition is a key factor when evaluating biodiversity evolution due to external factors such as climate change and natural disasters (Hologa et al., 2021). Another important application for tree identification is to evaluate the survival rate of seedlings, which is vital to assess the efforts of afforestation, identifying seedlings across several seasons is a difficult task, given the fact that each individual tree crown needs to be identified in a complex vegetation environment (Guo et al., 2021). Forest inventory and mapping are crucial for forest managers, to ensure the preservation of the different habitats (Neuville et al., 2021).

### 2.6 Tree structure

**Figure 2:**
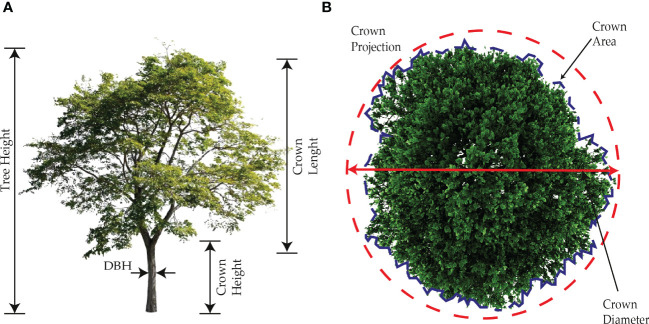
Tree structure parameters used to assess forestry health. In **(A)** are shown the parameters from a frontal view, **(B)** shows the parameters from an aerial point of view, focusing on the tree crown.

Tree and forest structure is related to forest biodiversity and productivity (Bohn and Huth, 2017). Tree structure identification is related to the measurement of parameters that help to characterize both individual trees and forests alike. The most common parameters used to characterize tree structure are tree height, diameter at breast height, basal area, total stem volume, crown cover, crown height, and crown area (Lin and Herold, 2016; Shin et al., 2018; Fraser and Congalton, 2021b; Guo et al., 2021; Hologa et al., 2021; Neuville et al., 2021; Terryn et al., 2022). These parameters are strong indicators of forest vigor and forest health when facing stress due to climate change (Fraser and Congalton, 2021b). Tree structure is essential in studies such as forest meteorology, botany, and ecology (Lin and Herold, 2016; Terryn et al., 2022). There is also a correlation between tree structure and the exchange of energy, carbon, and water between the forest canopy and its environment. **Figure 2** indicates the most common parameters that are used to assess forest structure

### 2.7 Tree classification

In the assessment of forested areas, tree species present distinctive traits such as textural characteristics and a specific spectral reflectance; these traits allow researchers to identify each tree species (Zhang C. et al., 2021). One of the purposes of tree classification is to know which tree species are able to regulate temperature and relative humidity in a certain environment, a fact that helps to better understand forested ecosystems (Liu et al., 2021; Zhang C. et al., 2021). Tree species classification is a crucial research topic for effective forest management (Onishi and Ise, 2021). Nevertheless, the most predominant factors that prevent a well-performed tree classification procedure are due to the diversity of tree species and the complexity of land (Zhang C. et al., 2021). Thus, gathering this data usually requires carrying out *in situ* measurements from sample plots and extrapolating to larger scales (Hell et al., 2022). Overall, this shallow or deep mapping is processed by hand-crafted features or specialized methods (Mäyrä et al., 2021). Currently, there are some new developments in this field, where researchers have introduced novel techniques related to computing various vegetation indices and textural features (Mäyrä et al., 2021), machine learning-based models, deep learning methods to extract tree features (Liu et al., 2021; Onishi and Ise, 2021) and the full use of forest spectral information (Zhang C. et al., 2021). Moreover, sensors and platforms used for this task, have become more specialized in order to capture enough information to accurately assess the type of tree (Mäyrä et al., 2021; Onishi and Ise, 2021; Zhang C. et al., 2021).

### 2.8 Crown fuel estimation

Several forest fire prediction studies rely on empirical models (Barber et al., 2021) using site-specific information on climate, topography, and fuels (Arkin et al., 2021). This information is strongly important for fire-prone countries in order to predict the impact of fire in certain scenarios. Fuel management programs (Wells et al., 2021) have been considered to reduce fire risk. The behavior of wildfires can be predicted by Crown Fuel Estimation (CFE). CFE is the assessment of fuel hazard layers. CFE is the assessment of fuel hazard due to the spatial arrangement of vegetation elements (branches, leaves, etc.); thus CFE helps researchers assess the severity of wildfires (Hillman et al., 2021), this task plays a key role since canopy fuels are the primary fuel layer of initiation and spread of crown fire (Arkin et al., 2021). It is worth mentioning that an accurate CFE can infer in the total or partial wildfire mitigation (Hillman et al., 2021; Wells et al., 2021). However, to completely assess the risk of wildfire; models including not only CFE but other tree structure parameters are needed; for example, the measure of live crown base height is critical this metric helps to estimate the likelihood of fire propagating from the surface into tree crowns (Arkin et al., 2021).

## 3 Hardware for remote sensing applications

In remote sensing applications, hardware fulfills vital roles in the data acquisition process, and choosing the correct sensors is critical to the success of the desired task (Müllerová et al., 2021). This section describes the different sensors, imaging systems, and platforms used in the reviewed articles.

### 3.1 Sensors

**Figure 3:**
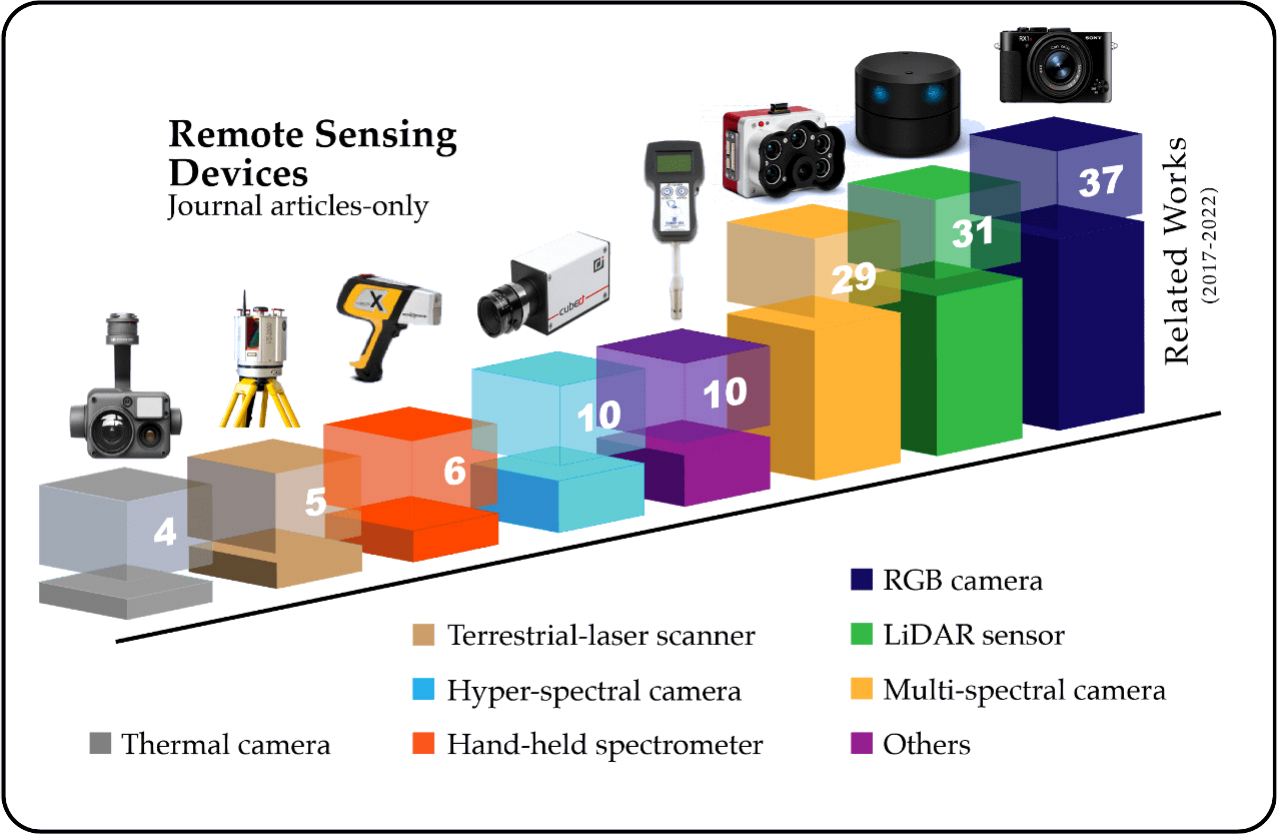
Most common sensors used for forestry health assessment, each column represents the number of articles that used each sensor in the data collecting process.

Remote sensing platforms include various kinds of sensors for gathering information about the environment. The most common sensors for forestry health assessment include the following: Visible Light Cameras (RGB Cameras), multi-spectral cameras, hyper-spectral cameras, thermal cameras, Laser imaging Detection and Ranging (LiDAR) systems, terrestrial laser scanning systems (TLS), and other common sensors. This section will discuss the working principle of the most common sensors in remote sensing for forestry health assessment. **Figure 3**, contains a visual representation of the most common sensors used for forestry health assessment.

#### 3.1.1 RGB cameras

**Figure 4:**
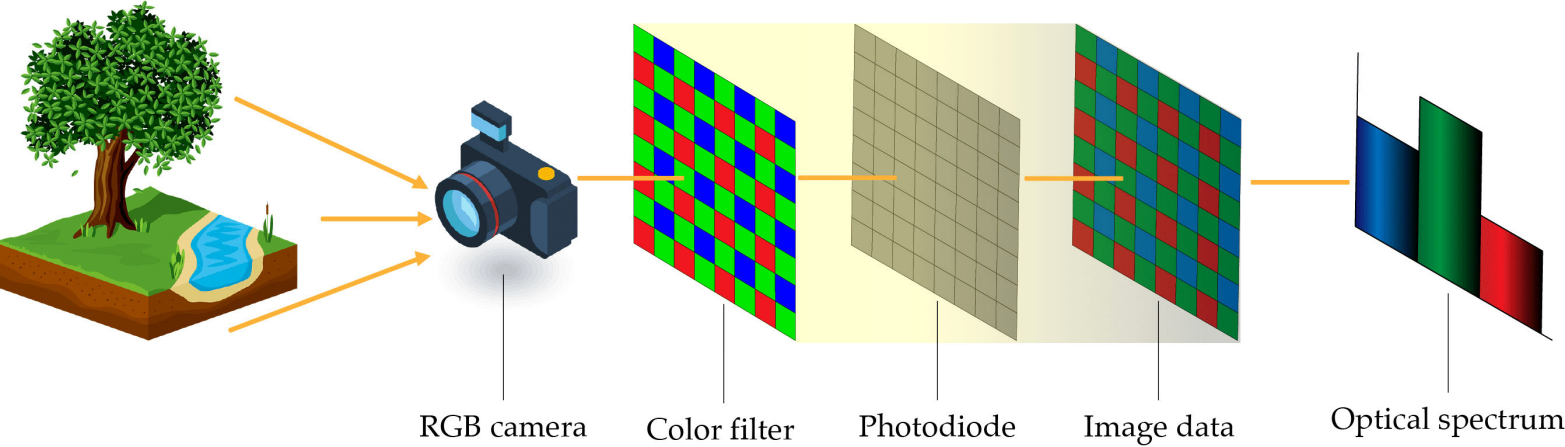
RGB camera working principle: a typical image processing system.

RGB cameras capture spectral information in visible light (400-700 nm), which is the same spectrum perceived by the human eye (Idrissi et al., 2022), the working principle of this kind of camera is visualized in **Figure 4**. These cameras are designed to represent the real colors of objects and nature using trichromatic red (620 - 750 nm), green (495-570 nm), and blue (450 -495 nm) wavelengths. Overall, RGB cameras provide two-dimensional images (Lin et al., 2022), and their performance tends to decrease in the presence of adverse atmospheric conditions (fog, haze, heat waves, etc.). The quality of an RGB camera is expressed in megapixels, which determine the number of pixels (i.e. length x height) of a static photo (Linhares et al., 2020). RGB cameras have been used for the study of vegetation indices based on RGB information (Ilniyaz et al., 2022; Talavera et al., 2022; Yang et al., 2022), forest canopy mapping and modeling (Nasiri et al., 2022; Suwardhi et al., 2022; Trencanová et al., 2022), tree identification and characterization(Onishi and Ise, 2021), and among others.

#### 3.1.2 Multi-spectral cameras

**Figure 5:**
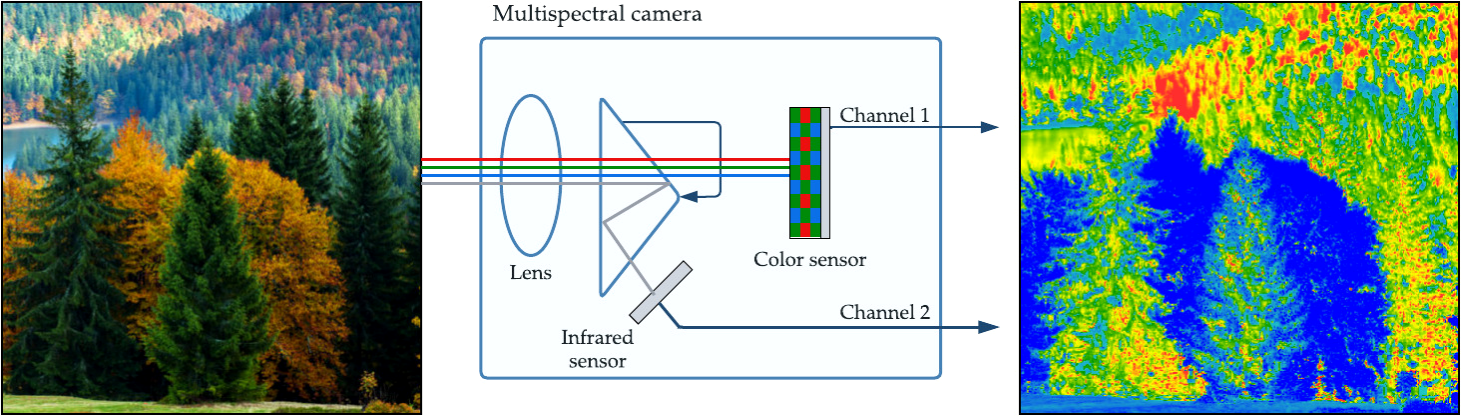
Multi-spectral imaging: camera structure and a sample of spectral forestry images.

Multi-spectral cameras collect color data and spectral monitoring. They capture two or more bands in the visible and invisible spectrum (Akkoyun, 2022). These cameras are able to cover parts of the infrared and ultraviolet regions. The most common wavelengths for these cameras are the Near-infrared wavelength (NIR) and red-edge wavelength from the infrared spectrum. Likewise, multi-spectral cameras hold a sensitive area detector used in conjunction with a series of specific waveband filters or a waveband tunable light source (Ramirez et al., 2022). The working principle of a multi-spectral camera is shown in **Figure 5**, with a visual representation of an image expected from this camera. In forestry health assessment, multi-spectral cameras have been used to obtain spectral indices and the derived applications as seen in previous sections.

#### 3.1.3 Hyper-spectral cameras

**Figure 6:**
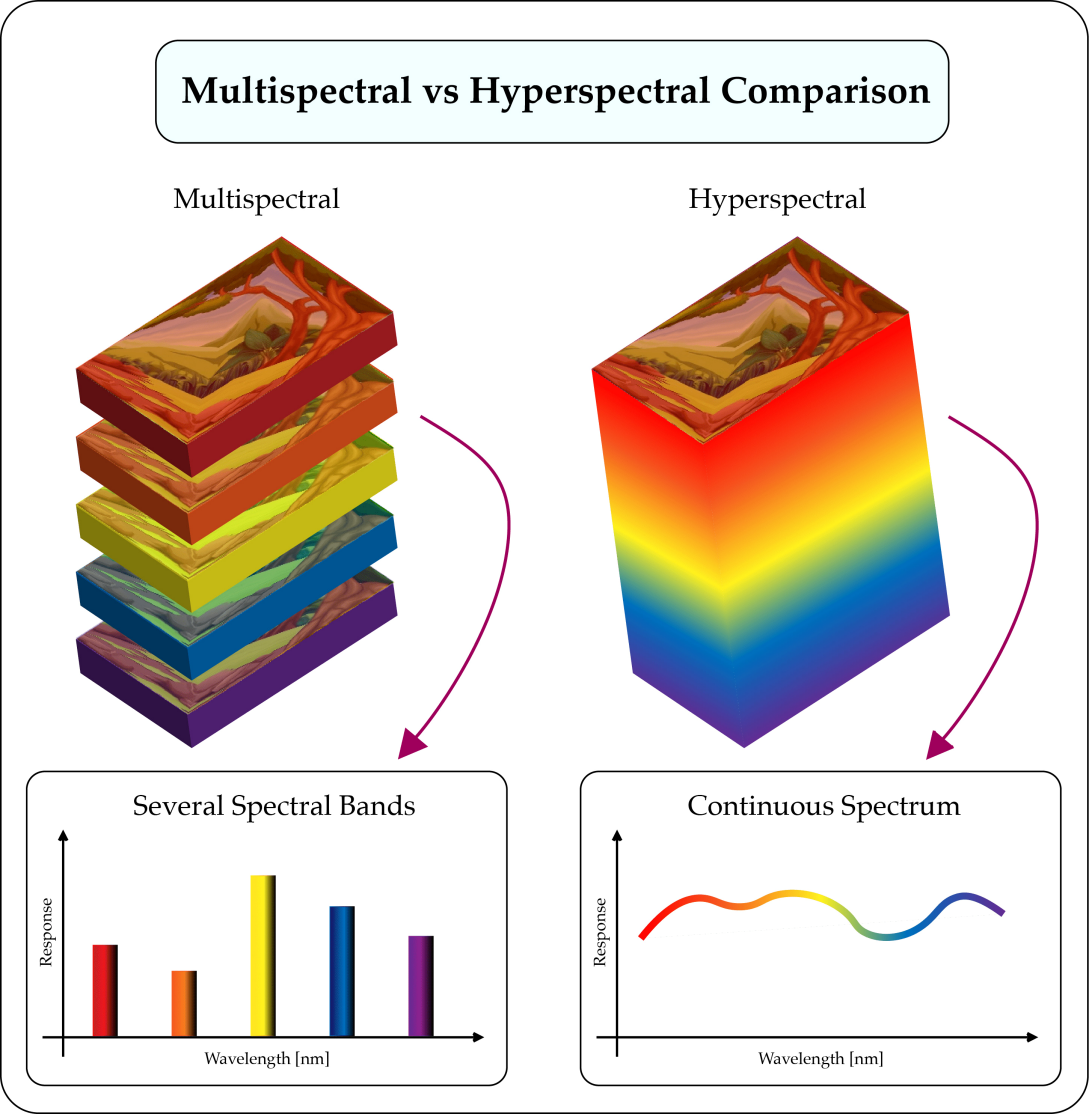
Comparison between Multi-spectral and Hyper-spectral camera operation. The multi-spectral camera presents a discrete and reduced number of bands, however, the hyper-spectral camera presents a continuous spectrum that ranges from wavelengths of 5 to 20 nm.

Hyperspectral sensors capture the radiation emitted by bodies in many bands, that go from hundreds up to thousands of wavelength bands, with narrower bandwidths than multi-spectral cameras (from 5 to 20 nm). Other sensors like RGB or Near-infrared (NIR) cameras only capture a minor number of bands (three in the case of RGB) (Adão et al., 2017). A comparison of multi-spectral and hyper-spectral cameras is shown in **Figure 6**, the main difference is that the hyper-spectral captures a continuous representation of the light spectrum, given the fact that it collects the reflectance in narrow bands; but the multi-spectral cameras only capture the reflectance in a selected number of bands.

Hyperspectral cameras have been used in forestry, to obtain new VIs to predict vegetation features such as leaf nitrogen content (Raj et al., 2021), chlorophyll, and other photosynthetic plant traits (Watt et al., 2020). Mapping forest hyperspectral characteristics have been performed as well (Weinstein et al., 2021). The main advantage of using hyperspectral cameras is the increased number of wavelengths, thus more information is gathered about the environment, however, the models created using this information might be overfitted and thus not usable in general cases (Lee et al., 2004).

#### 3.1.4 Infrared cameras

Infrared cameras are a specific type of sensor that captures the infrared radiation that is emitted by all bodies with a temperature above absolute zero. The range of wavelengths that is captured by these sensors depends on the nature of each one, but common wavelengths are Short-wave Infrared (SWIR) that ranges from 700 to 1400 *nm*, Mid-wave, infrared (MWIR) from 3000 to 5000 *nm*, and Long Wave infrared (LWIR) that ranges from 8000 to 14000 *nm* (Gade and Moeslund, 2013), these sensors are also known as thermalcameras in the reviewed studies (Xu et al., 2018; Cheng et al., 2022).

**Figure 7:**
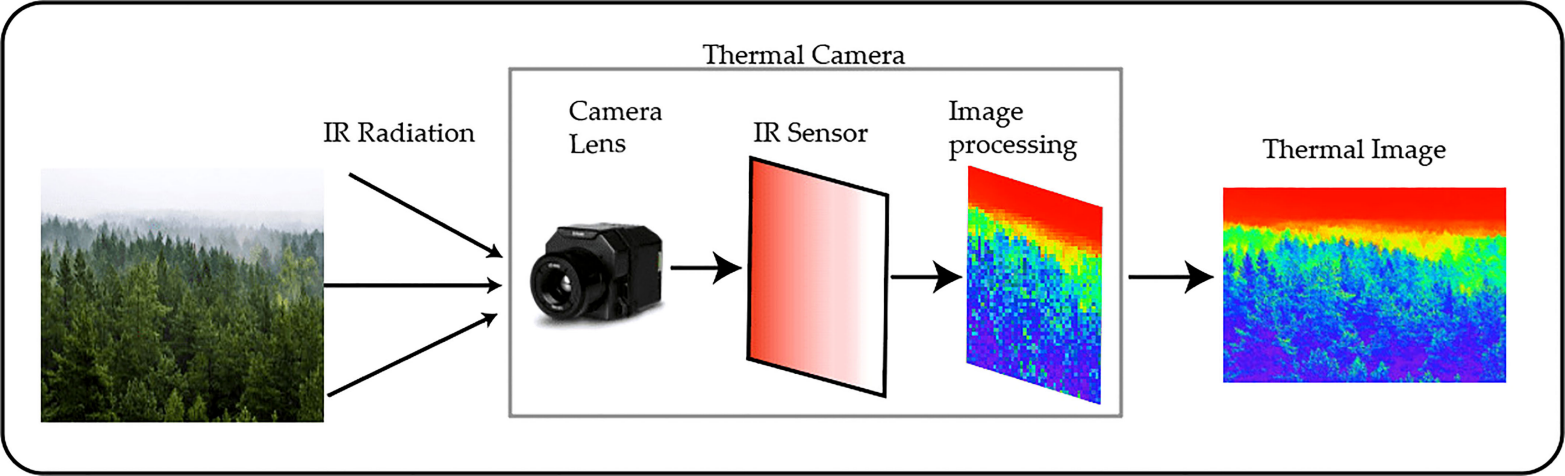
Internal Structure and expected forestry image from a thermal camera.

**Figure 7** shows the common structure of a thermal camera used in remote sensing applications. These sensors have been used in forestry health assessment to create thermal mappings that are coincident with RGB mapping information (Webster et al., 2018). Other applications include the use of thermal indices to predict soil moisture (Cheng et al., 2022) and for phenotyping (Xu et al., 2018).

#### 3.1.5 LiDAR sensor

**Figure 8:**
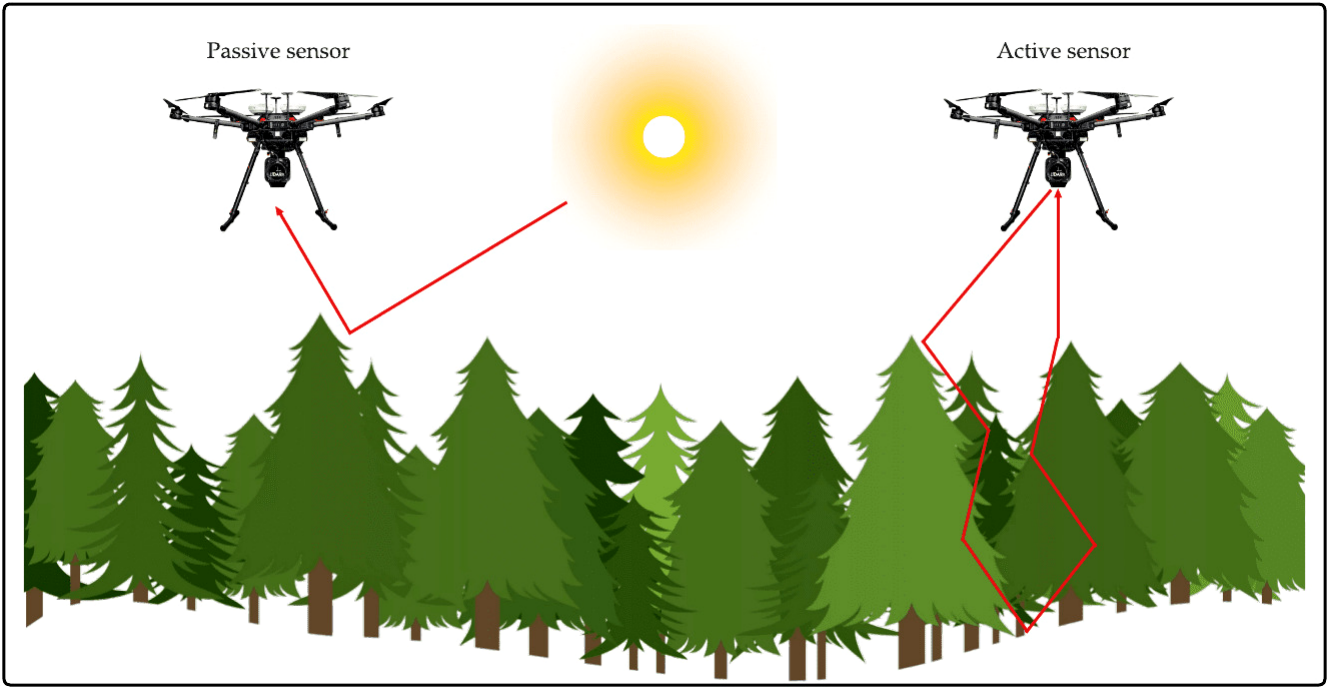
Differences between Passive sensors and Active sensors.

LiDAR (Light Detection And Ranging or Laser Imaging Detection and Ranging) sensor is a device widely used for remote sensing. It is considered an active device due to its light emission and detection (See **Figure 8** for comparison with passive sensors). Moreover, this sensor has two key elements to gather and analyze data: photodetector and optics. The principle of LiDAR is to emit laser light towards an object on the Earth’s surface and compute how long it takes to return to the LiDAR emitter, this definition holds for an airborne-based LiDAR system (Khairul and Bhuiyan, 2017).

The LiDAR point cloud is useful for obtaining physical information about the surveyed area, the 3D measurements can be used for generating terrain models, then by processing the LiDAR point cloud information digital terrain models and digital elevation models can be retrieved by thresholding the altitude of each point and discerning which point can be considered from terrain or from the top tree crowns. With this information, elevation models are easily obtained by subtracting the digital elevation modelsand digital surface models surface models (Hologa et al., 2021). LiDAR point clouds are also useful for obtaining geometric features of vegetation as slopes or texture information; these metrics are then used as input data for machine learning models with various tasks for example (Hologa et al., 2021), uses geometric descriptions of vegetation obtained from a point cloud to perform tree classification, a similar approach is done in (Hell et al., 2022). Due to the resolution that the LiDAR point cloud is capable of generating, individual trees can be identified, and thus tree metrics can be directly computed. In (Vizireanu et al., 2020; Neuville et al., 2021), DBH is estimated based only on LiDAR retrieved data, other forest attributes estimated by LiDAR cloud points are canopy cover (Cai et al., 2021), which can be derived through the density of vegetation points, this metric is also used to predict biomass near rivers (Resop et al., 2021), and with the purpose of determining crown fuels (Suwardhi et al., 2022). Morphological features derived from LiDAR point cloud can be key factors to determine and differentiate between alive trees and snags or deciduous and evergreen trees, this study is done by Stitt et al. (2022). The use of LiDAR has helped researchers investigate the following: tree modeling (Suwardhi et al., 2022), biomass estimation (Torre-Tojal et al., 2022), and tree classification (Hell et al., 2022) among others.

#### 3.1.6 Terrestrial laser scanning systems

**Figure 9:**
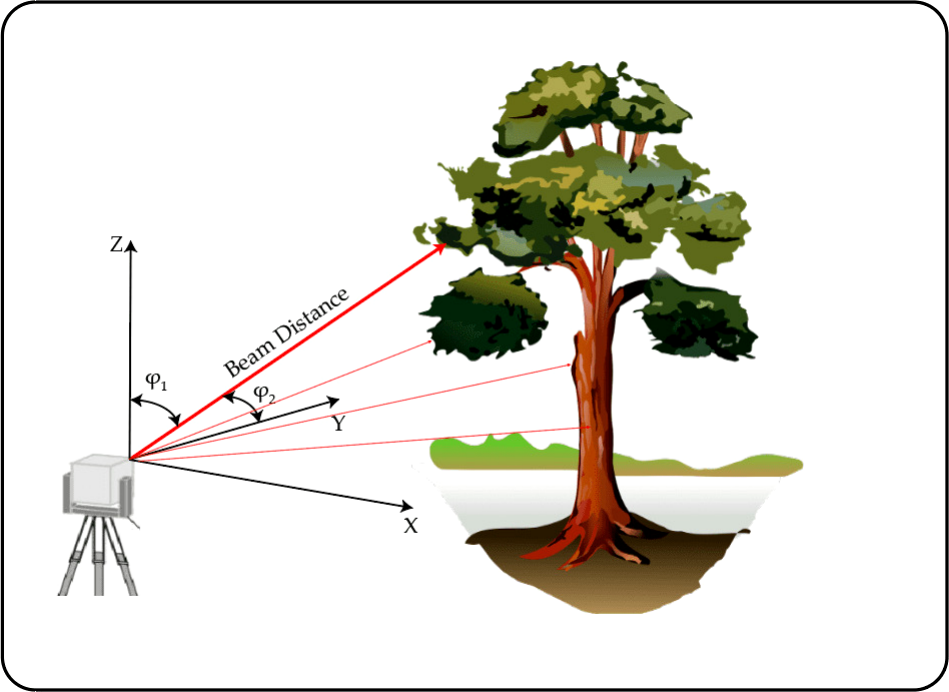
TLS sensor variables needed for obtaining 3D cloud points.

Terrestrial laser Scanning Systems (TLS) are instruments used to obtain three dimension observation of the surface of objects. It uses LiDAR sensing to obtain the distance from the surface to the sensor, and precise angular measurements to obtain 3D information from the objects. TLS systems are capable of reconstructing an area with high precision in the order of millimeters (Liang et al., 2016). A representation of the TLS and its measurements are shown in **Figure 9**. In forest health assessment, TLS systems are used to determine tree features and structure (Miraki and Sohrabi, 2021; Terryn et al., 2022; Yang et al., 2022), and to estimate crown fuel and fuel hazard (Hillman et al., 2021).

#### 3.1.7 Handheld spectrometer

A handheld spectrometer is a device that is capable to retrieve the spectrum emitted by a body in many wavelength bands, the same as a hyper-spectral camera, but this one is portable and operated by hand. Another difference is that a hyperspectral camera captures many pixels, and the spectrometer only captures a single point. The main application for this device is to obtain samples of an object that will serve as ground truth for mass data obtained with a camera or by other means. Handheld spectrometers have been used to gather information to estimate leaf water content (Li et al., 2021), to monitor the chlorophyll response to droughts (Raddi et al., 2021), and to perform tree recognition based on hyper-spectral features (Yang and Kan, 2022).

#### 3.1.8 Others

There are other kinds of sensors used for forestry health assessment. For instance, an ANAFI camera (Ribas Costa et al., 2022), wireless sensors (Yang et al., 2022), a thermocouple (Yao et al., 2021), and a SPAD-502 meter (chlorophyll meter) (Yao et al., 2021; Narmilan et al., 2022). These sensors are used for very specific scenarios, such as measuring chlorophyll in a single leaf, and thus are not considered for further revision in this review.

### 3.2 Remote sensing platforms

This section presents a brief review of the most common remote sensing platforms; highlighting their advantages, disadvantages, and applications; for a most extensive review on the topic, see (Omasa et al., 2006; Ashraf et al., 2011; Pajares, 2015; Toth and Jó´zków, 2016; Zhang K. et al., 2020; Chamola et al., 2021; Zhao et al., 2022).

**Figure 10:**
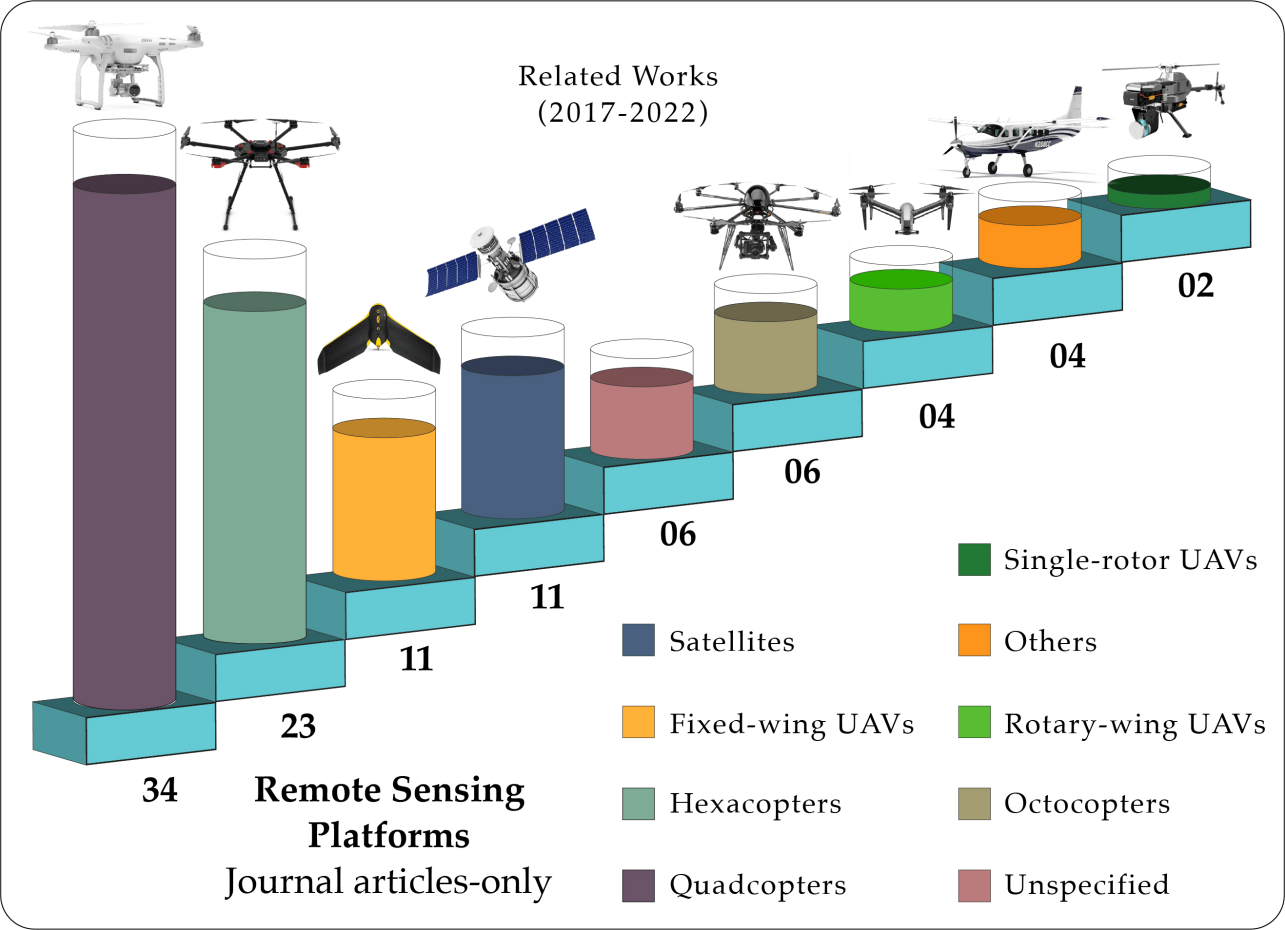
Distribution of the most common UAV platforms in the reviewed journal articles.

**Figure 11:**
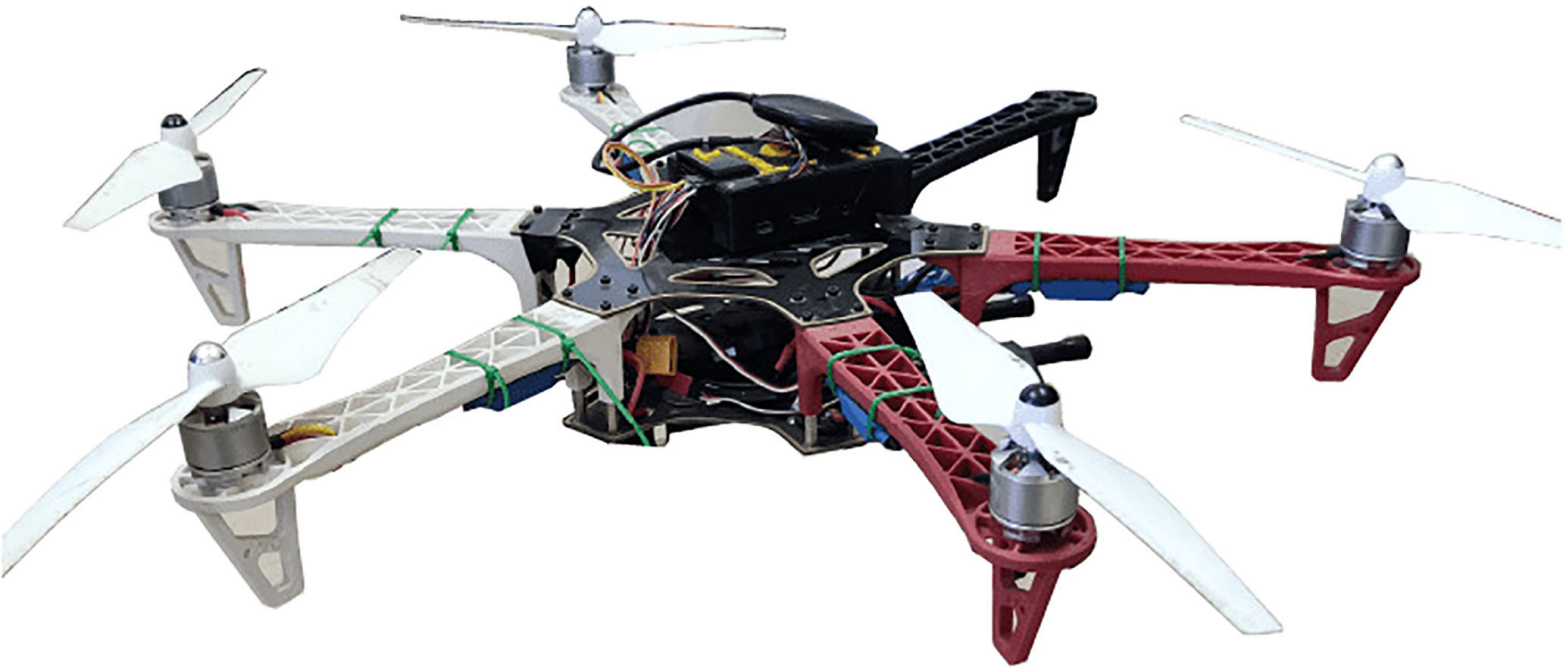
A remote sensing platform mounted on a hexacopter.

Remote sensing platforms are understood as the platforms that physically carry the different cameras and sensors used for the assessment of forestry health. There are two major groups of platforms that are identified: Unmanned Aerial Vehicles (UAVs) and satellites. **Figure 10**, summarizes the number of appearances that the different remote sensing platforms have in the reviewed articles. **Figure 11** shows a remote sensing platform using a UAV.

#### 3.2.1 Satellites

Satellites are commonly used for remote sensing purposes (Zhao et al., 2022). These devices are aimed at gathering data from Earth using imaging sensors. Satellites tend to capture electromagnetic radiation in the microwave, ultraviolet, and visible wavelengths reflected by the Earth’s surface (Ashraf et al., 2011). Overall, a remote-sensing satellite is able to take 4-5 photos with different types of color filters, evidently, these color filters help to better assess vegetation features such as soil, leaves, stems, tree crowns, under/over the canopy, and so on.

Satellites carry onboard high-resolution microsatellite cameras (HR-250 and Raptor imagers) with advanced electronic detectors known as CCDs (Charge-Coupled Devices). These devices not only allow them to be more sensitive than a film but also convert the multispectral photographs into electronic signals for further study (Zhang K. et al., 2020).

According to the literature reviewed, Sentinel 1 and 2 (Huo et al., 2021; Nasiri et al., 2022), Landsat-8 (Rodríguez et al., 2021), Worldview-2 (Becker et al., 2018), Triplesat (Fakhri et al., 2022) are the most prominent satellite platforms used to assess forestry health.

#### 3.2.2 UAVs

Unmanned Aerial Vehicles are the most common platforms in remote sensing applications for forestry health assessment. The typical UAV for remote sensing is an electric-propelled air vehicle, with a navigation system and communication system, and a sensor for remote sensing (Toth and Józków, 2016). The navigation and flight control systems are composed of various onboard sensors in the UAV, the main ones are: Global Positioning System (GPS), an Inertial Measurement System (IMU), and Micro-Electromechanical System (MEMS) (Toth and Józków, 2016). The other components of the remote sensing platform are the sensors needed for the data acquisition process, the most common sensors in remote sensing applications are the ones mentioned in section 3.1.

There are different kinds of UAVs, and according to their configuration, they offer different features such as higher payload capability, longer flight capacity, and better maneuverability among others. We have identified the following classes:

##### 3.2.2.1 Single-rotor

Single-rotor UAVs are formed by a single rotary wing, they are a minority compared to other remote sensing platforms. Since they only present a single rotor they present a much higher power efficiency compared to multi-rotor UAVs, they are also used for carrying heavy payloads (Chamola et al., 2021).

##### 3.2.2.2 Multi-rotor

Multi-rotor UAVs are the most versatile and have been used in a wide range of operations. This group includes quadcopters, hexacopters, and octocopters. The main advantages of using these UAVs are their commercial availability and affordability, the ease of maneuverability, they don’t need a platform to take off, meaning that they can take off and land on any surface; so they are preferred for research purposes. The arrangement of multiple rotors provides the UAV with better stability making them ideal for imaging purposes (Toth and Józków, 2016; Chamola et al., 2021).

##### 3.2.2.3 Fixed-wing UAV

These UAVs present a stationary wing, similar to a plane, the advantage of using a fixed-wing is that lift forces are lower compared to rotary wing UAVs. Since they are similar to a plane they need some area for the takeoff and eventual landing. The main advantage of fixed-wing drones is that they can fly for longer periods of time, cover larger areas, and can carry heavier payloads (Chamola et al., 2021).

##### 3.2.2.4 Aircraft

Forestry studies have evoked their efforts to incorporate remote sensing aircraft into the dynamics of forest surveys and data collection. Aircraft remote sensing platforms rely heavily on onboard sensors to leverage their advantages associated with flexible use and high spatial resolution. In addition, images captured from the aerial inspection can be used for rapid analysis in different seasons of the year (Omasa et al., 2006).

## 4 Machine learning techniques used in forestry health assessment

Machine learning is a set of algorithms that require the computer or machine to infer and extract patterns from raw data (Goodfellow et al., 2016); the effectiveness of machine learning heavily depends on the representation of the data fed to the model. These algorithms can be used for regression tasks, which implies predicting a number from a set of input data; classification problems can also be accomplished by machine learning, in this case, the algorithm predicts that the data representing a feature belongs to a predefined class.

Learning is a key concept in machine learning, it can be performed in these ways:

### 4.1 Supervised learning

In supervised Learning algorithms, the dataset containing features also contains a number or a label that is the expected output from the input features. In this case, the machine learning algorithm needs to infer which is the relation between the set of features and the expected output, then apply these found relations in a set of testing data (Goodfellow et al., 2016).

### 4.2 Unsupervised learning

In these algorithms, the dataset contains a set of features and the algorithm learns properties about how the data is structured, a common task performed by unsupervised learning is to recreate the probability distribution that generated the dataset; another common function is to group data into clusters with similar characteristics (Goodfellow et al., 2016).

### 4.3 Metrics

It is important to measure how the machine learning algorithm is performing its task, thus it is important to describe the most common metrics to quantitatively evaluate the algorithm’s performance. The following are the most used metrics for classification purposes:

#### 4.3.1 Accuracy

It can be defined as the ratio between the number of correct predictions and the number of total predictions made by the model (Flach, 2019), it can be calculated with Eq. (1)


(1) $$Accuracy=\frac{TP+TN}{TP+TN+FP+FN}$$

Where TP, TN, FP, and FN stand for True Positive, True Negative, False Positive, and False Negative respectively.

#### 4.3.2 Precision

It is the ratio between correct positive predictions and total prediction, it indicates the proportion of how many correct predictions the model yields, it is calculated with Eq. (2)


(2) $$Prec=\frac{TP}{TP+FP}$$

#### 4.3.3 Recall

It measures the ratio of correct positive predictions and the total predictions, it is obtained with Eq. (3)


(3) $$Rec=\frac{TP}{TP+FN}$$

#### 4.3.4 F1 Score

It is a metric that combines both Precision and Recall, it is useful when the classes in a dataset are unbalanced, and it is computed with Eq. (4)


(4) $$F_{1}=2\cdot \frac{Prec\cdot Rec}{Prec+Rec}$$

#### 4.3.5 Root mean square error

It is a measure of the error between the predicted output of the model and the real output of the model. This metric is used for evaluating regression models. It is computed with Eq. (5)


(5) $$RMSE=\sqrt{\frac{{-}_{i=1}^{N}||y(i)-\hat{y}(i){\|}^{2}}{N}}$$

#### 4.3.6 Correlation factor (*R* ^2^)

It is a number that indicates if there is a correlation between two variables, in regression models it is a metric that helps to understand if the output of the model is correlated with the input. It ranges from 0 to 1, where 0 indicates that there is no correlation between the variables and 1 that there is a high correlation.

**Figure 12:**
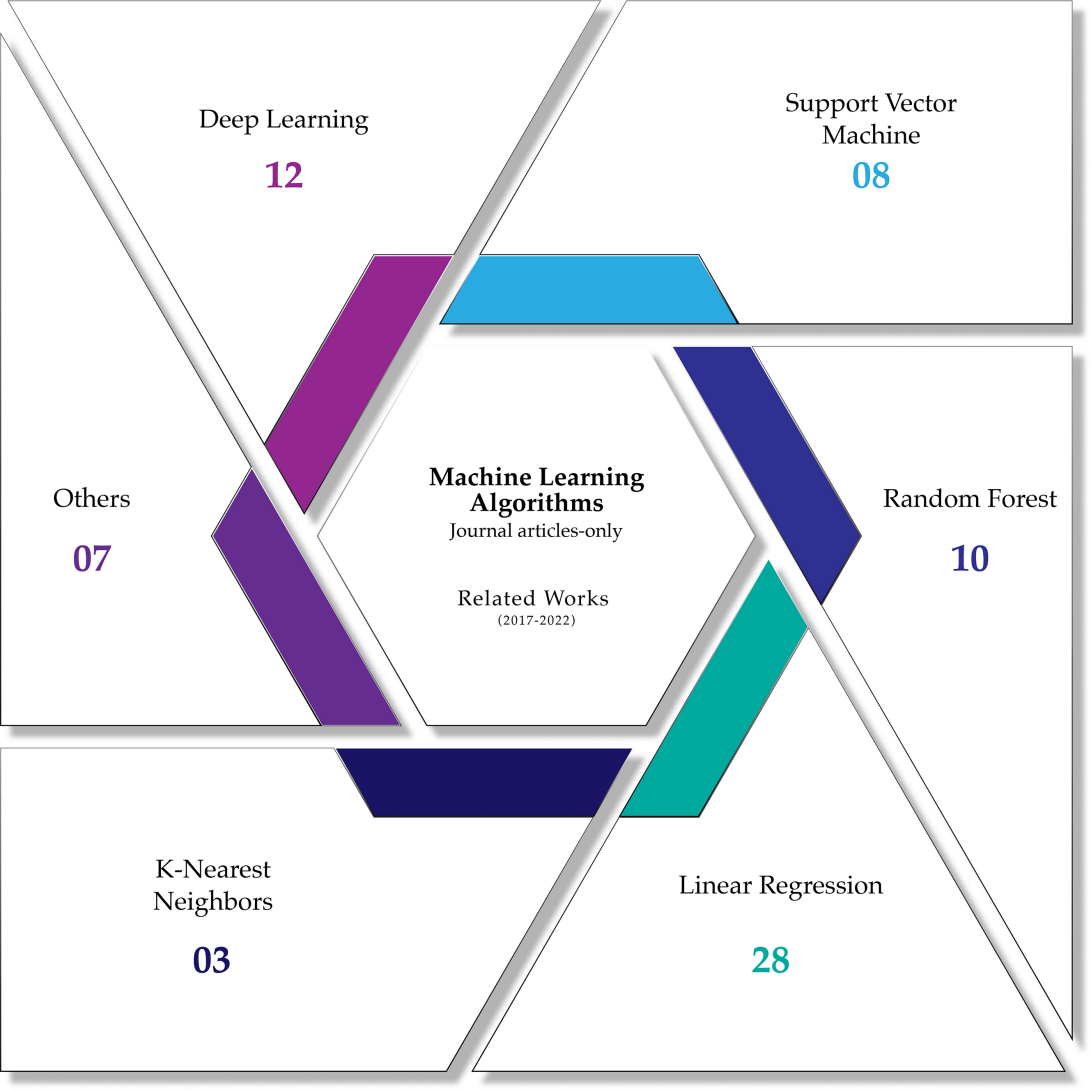
Distribution of the most common machine learning algorithms for forestry health assessment in the reviewed journal articles.

**Figure 13:**
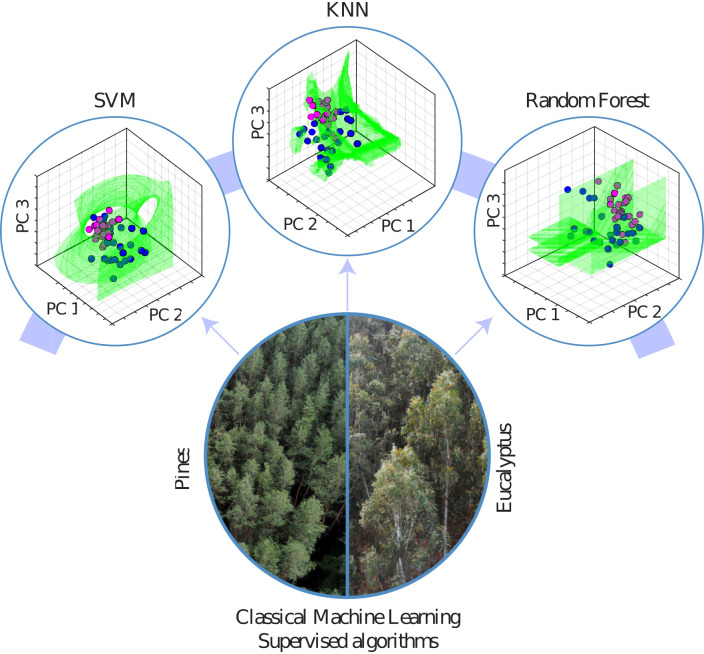
A comparison between the most common machine learning methods, and how the space is divided to generate different classes.

With the previous remarks, the section continues describing the most common machine-learning techniques used in the reviewed articles for the assessment of forestry health and the most critical results supported by quantitative metrics, the discussed algorithms in the section are: Linear Regression, Random Forests, Support Vector Machines, K-Nearest Neighbours, deep learning approaches and other not common machine learning techniques. **Figure 12** shows the most common ML algorithms used in forestry health assessment in articles from the last five years. **Figure 13** shows a visual representation of how three of the most common ML methods divide the search space for classification purposes.

### 4.4 Linear regression

Linear Regression is one of the most common algorithms in machine learning, for predicting results. Using an optimization process,linear regression determines the appropriate equation that maps the input features with the expected output (Goodfellow et al., 2016). Linear regression has had a wide range of applications. It has been used to find the correlation between the data derived from TLS and airborne LiDAR; the study presented by Hillman et al. (2021) demonstrated that estimations of canopy volume have a strong correlation between the data from LiDAR and TLS which achieved a value of 0.96, herein the ground-truth is the value obtained from the TLS sensors, however other tree structure parameters such as canopy base height achieved only a correlation of 0.794. In other studies canopy height volume reached a correlation of only 0.394, thus it is not suitable for predicting crown fuel (Shin et al., 2018), similar experiments were conducted by Arkin et al. (2021). For predicting the moisture of leaf fuels, multi-spectral VIs were used as input data for regression models, however, the correlation factor reached 0.435, thus more studies are needed for practical implementations for this model (Barber et al., 2021).

Other vegetative problems are investigated using linear regression models. Resop et al. (2021) studied the correlation between vegetation metrics, the distance from water sources, and seasonal variation; the results show that there is no correlation between the distance to the water stream and canopy height and vegetation density. Using multi-spectral VIs, regression models have been used to predict biomass in the tidal marsh; the best VI was ExG however the correlation index only reached 0.376 (Morgan et al., 2021). In coastal wetlands, the correlation between above-ground biomass and flood depth was studied, and the regression models follow a Gaussian distribution with a correlation factor of 0.54 (Yan et al., 2022). Xu et al. (2022) studied the correlation between tree diversity and spectral indices. The correlation value was 0.6; thus VIs could be used for tree classification purposes.

Estimating the correlation between tree features and point cloud LiDAR data information, in the work presented by Fraser and Congalton (2021a) RGB and LiDAR-derived metrics of DBH and crown radius were studied in a coniferous forest. The results show a correlation of 0.392 and RMSE which equates to 30% of the total error. Fan et al. (2020) created tree models derived from LiDAR point clouds, and then structure metrics were calculated, the predictions were correlated with the ground truth collected *in situ*, and the linear models achieved a correlation of more than 0.9 for DBH, tree height, and crown volume. In the article by Imangholiloo et al. (2020), tree height was estimated using LiDAR metrics, such as point density, in leaf-on and leaf-off seasons, and the correlation factor achieved 0.98. A similar study conducted by Puliti et al. (2019), compared tree height, stem volume, and basal area; from data obtained via different aerial methods (UAV, and manned aircraft); the results show correlation values in the range of 0.64 and 0.73. Another study combined RGB images and LiDAR metrics to predict tree height and DBH in a eucalyptus forest, combining both metrics as input data for the model achieved a correlation of 0.94 (Liao et al., 2022). Xu et al. (2021) developed a remote sensing platform and the method of validating its data was to find the correlation of tree structure parameters with the ground truth found in the field, this study also contemplated the creation of thermal and multi-spectral VIs.

Leaf area index (LAI) is another parameter that can be predicted using LiDAR metrics and linear regression models. In the work by Tesfamichael et al. (2018) the highest correlation value was 0.83; however, this model used several metrics as input data; a simple model using only two metrics achieved a correlation of 0.63 but the simplicity of the model was considered an advantage. A similar study using RGB point clouds for calculating LAI was conducted by Lin L. et al. (2021), and the models achieved a correlation of 0.92. Miraki and Sohrabi (2021) estimated LAI from RGB images and terrain model descriptors as input data, but the correlation was only 0.42, in the same study canopy height was also estimated, and using linear regression models the correlation achieved was 0.84. The study presented by (Qiao et al., 2022) also considered morphological features from the soil and the vegetation to improve the prediction of LAI, achieving correlation values of 0.93 but it depends on the growth stage of the vegetation. Water and transpiration models are also associated with LAI and canopy volume; Aboutalebi et al. (2019) estimated these parameters using information derived from airborne LiDAR and multi-spectral cameras; the LAI derived by machine learning achieved correlations of 0.7.

Predicting the chlorophyll changes in response to environmental changes has been explored with the aid of regression models. In the study presented by Raddi et al. (2021), using hyper-spectral indices and multi-spectral indices; leaf chlorophyll content in textit Quercus Robur, Quercus Pubescens, and *Quercus ilex* was estimated with the aid of linear regression models; using both kinds of indices achieved a correlation of 0.97 in both cases, thus providing an excellent alternative to assess drought responses using the change of chlorophyll content as an indicator. Zhuo et al. (2022), conducted a similar study to predict chlorophyll content, however, it considered the effect of mixed vegetation in wetlands for the computation of the spectral indices, in this case, the model reached a correlation of 0.82. (Kopacková-Strnadová et al., 2021) presented a study aimed to predict photosynthetic pigments in coniferous Spruce forests, using multi-spectral VIs; however, the researchers showed that information from the growth stage of the forests is needed since the spectrum from two years’ leaves was the only VI that reached a correlation factor of 0.52 in a linear regression model. Watt et al. (2020) conducted a similar procedure but with the purpose of predicting nitrogen and phosphorus. Using hyperspectral VIs, regression models were trained and the predictor for both P and N achieved correlations of 0.75 and 0.83 respectively. Other studies predicting chlorophyll in different ecosystems are done by Narmilan et al. (2022) and Yao et al. (2021), with the purpose of evaluating soil respiration; estimating nitrogen can be achieved with regression models and RGB indices (Lu et al., 2021).

Problems related to moisture content, in general, can be performed using linear regression. In the work presented by Li et al. (2021), leaf water content estimation was performed using hyper-spectral VIs in various growth stages of vegetation reaching a correlation factor of 0.9 with the appropriate VI. Regression models were also used to assess water evaporation models and trace element uptake by trees growing on red gypsum landfill (Malabad et al., 2022). Cężkowski et al. (2020) used thermal indices used to predict various indicators of water stress in wetlands (soil moisture, chlorophyll content, and photosynthetic active radiation (fAPAR)), the correlation factors for soil moisture and fAPAR were of 0.62 and 0.70 respectively, thus the index could be an indicator of water stress.

### 4.5 Random forest

Random Forest is a machine learning method that combines multiple tree classifiers. Each tree is tested with a random input vector, which leads to selecting the most significant features from the input data. Random Forest can be used for classification and regression problems (Breiman, 2001).

For classification purposes random forest has been used in conjunction with information derived from LiDAR point cloud and with multi-spectral indices derived from spectral imagery; this approach presented by Hologa et al. (2021) was used to perform individual tree classification in a mixed forested area, the trained random forest achieved an accuracy of 96% over eleven different tree species when combining both inputs from LiDAR and multi-spectral imagery. A similar approach was done by Fraser and Congalton (2021a), but in this case, due to the nature of the forest, the classification task using random forest achieved an accuracy of 85%, but the authors highlight the capability of random forest over traditional methods for tree delineation. Imangholiloo et al. (2020) used random forest for classification between coniferous and deciduous trees from information obtained by LiDAR. In the work presented by Miyoshi et al. (2020), the input data included hyperspectral multi-temporal imaging data to perform tree classification in a diverse tropical forest, even though the accuracy only reached 50%, the use of multi-temporal imaging improved previous approaches using random forests as classifiers, leaving the door open to future researches in the same field.

Fraser et al. Fraser and Congalton (2021b), performed a classification of forest stands in three different categories: healthy, stressed, and degraded trees; for this purpose, VIs from multi-spectral imagery were derived and they were used to train the RF model; the accuracy achieved a maximum of 71%, due to the fact that there is a high variation in the characteristics of each healthy tree.

Classification tasks are not only needed to differentiate between tree species. Another important task is to classify between live trees and dead trees, the reason being this ratio is important for assessing the response of the ecosystem to external disturbances; Stitt et al. (2022) used information derived from a LiDAR point cloud to classify different kinds of snags, the model achieved an accuracy of 77%, signifying that only LiDAR information is not enough to identify some characteristics of snags. In the work by (Shovon et al., 2022), the RF algorithm was trained to segment between alive and dead trees in forest stands with an accuracy of 89.4%, using as input variables tree height derived from LiDAR point clouds and RGB spectral indices.

Identifying forest structure can be achieved by using random forest, Yu et al. (2021) explored the feasibility of using multi-seasonal data from LiDAR and multi-spectral images to perform vertical forest structure classification. The results show that adding information from different seasons as input variables to the models increases its performance and its capability of reliably identifying the forest structure, even though the random forest was not the best algorithm according to the metrics presented.

Individual tree recognition can be accomplished by random forest. Guo et al. (2021), with the purpose of assessing afforestation models, trained random forests methods to recognize areas of interest that could potentially be identified as tree crowns, for this purpose several VIs were computed from RGB images and they were used as training data for the random forest algorithm; the individual crown recognition task achieved an accuracy of 92%, when using more than two input variables to train the model.

Random Forests were also used for regression purposes. In the work presented by Lou et al. (2021), the feasibility of predicting canopy chlorophyll content in marsh vegetation was evaluated using multispectral images from UAVs, and from satellite platforms including Landsat-8 and Sentinel-2. The predicted canopy from the random forest was validated with the real value through a linear regression achieving a correlation value of 0, 92. Villacrés and Cheein (2022) used random forests to retrieve spectral VIs from multispectral imagery essential for mapping moisture content, however, the results were unsatisfactory, and other regression methods were needed.

Biomass prediction using Random Forest was explored by Torre-Tojal et al. (2022), for this purpose, a LiDAR point cloud was obtained using a UAV; subsequently, digital terrain models and canopy models were reproduced. Some of the metrics obtained were height distribution, canopy cover, and canopy height. An analysis of the importance of those metrics was performed resulting in that the metrics related to the height of the trees were the most significant when describing biomass; using these variables the RF was trained, and the predicted result of the model achieved a correlation value of 0.7, improving previous estimations. Indices and aerial images from satellite platforms are also promising sources of data for prediction purposes, Nasiri et al. (2022) used Sentinel-2 derived Vegetation indices with the purpose of mapping canopy cover in forested areas using Random Forest Regression to predict the percentage of canopy according to the indices, the trained model achieved a correlation of 0.69, showing the potential of combining satellite platforms and random forest for mapping purposes. Sentinel-2 imagery was used to predict the biomass of fine fuels in dryland ecosystems, and the training of the random forest yielded a correlation factor of 0.63 over a six-year period, highlighting the potential of machine learning techniques for mass land estimation of fine fuels (Wells et al., 2021).

### 4.6 Support vector machines

A support vector machine is a method mainly used for classification purposes, the objective of the SVM is to find a hyperplane that divides in the “best way” two different classes of data. The “best way” refers to the fact that the distance between the hyperplane and each class is maximum (Goodfellow et al., 2016). The main advantage of SVM is that it uses a kernel function that assigns the input data to a higher dimensional space, where it is easier to find the hyperplane that separates two classes.

In forestry health assessment SVMs are used to perform classification and regression tasks. In (Mäyrä et al., 2021), SVMs are used to perform the identification of tree species, using as input vectors point clouds from LiDAR and images from hyperspectral cameras from the SWIR region with 288 bands. From the point clouds, individual tree segmentation was performed and the SVMs were trained. This study shows that there are no major errors in tree classification processes using SVM, achieving an accuracy of 82%; although this method is outperformed by deep learning approaches (Mäyrä et al., 2021), which achieved an accuracy of 87%.

The work by Blanco-Sacristán et al. (2021) uses SVM to perform segmentation in images based on RGB and multi-spectral images. Images were segmented based on their level of dryness, it is important for monitoring possible fire-prone lands. The accuracy reached 80% in most cases.

Tree structure classification has also been studied with the aid of SVM (Yu et al., 2021), predicting the tree structure in a densely forested area, for this purpose the authors used LiDAR and Multi-spectral point clouds to generate height models which were used as inputs to the SVM, in this case, the classification from the SVM was outperformed by other methods. SVMs are used to evaluate carbon models from tree parameters such as canopy height and DBH (McClelland et al., 2019).

The segmentation of ground points based on VIs can be considered as a classification algorithm, in this context Zhang Y. et al. (2021) used vegetation indices as input data for SVM with the purpose of classifying ground points and vegetation points in aerial images; this method achieved an accuracy of 94% using only two VIs as input.

As a regression technique, Support Vector Regressor (SVR) was used to predict tree structure parameters such as DBH, tree height, and volume using as input data high-density LiDAR point clouds (Corte et al., 2020). The results show that the errors in the prediction were lower when using SVR, compared to other algorithms such as RF or neural networks. Nasiri et al. (2022) processed VIs derived from Sentinel-2 information to model canopy cover, achieving significant correlation values of 0.64. A similar task was performed by Abdollahnejad and Panagiotidis (2020), but the tree classification was performed with inputs from multi-spectral VIs.

### 4.7 K-nearest neighbors

K-nearest Neighbors is a non-parametric machine learning technique, which means that the training does not generate the optimum parameters for a mapping function or plane. It simply is a function of the training data, in its simplest form, KNN computes the expected output value from a new input, by averaging the output from the K nearest neighbors in the training data of this new entry (Goodfellow et al., 2016).

The KNN algorithm was used to perform tree classification from hyper-spectral information. In the work presented by Yang and Kan (2020), the input vectors were information from hyper-spectral imaging, in this case, the KNN algorithm was the least effective algorithm. Tuominen et al. (2017) used KNN to estimate tree structures from the information gathered manually in plots and predict them in aerial photos, the results show that the error is below 30 percent. Another use of KNN algorithm is presented by Zhang Y. et al. (2021), the model was used to segment ground points from vegetation points, however, this model was outperformed by SVM.

### 4.8 Deep learning

**Figure 14:**
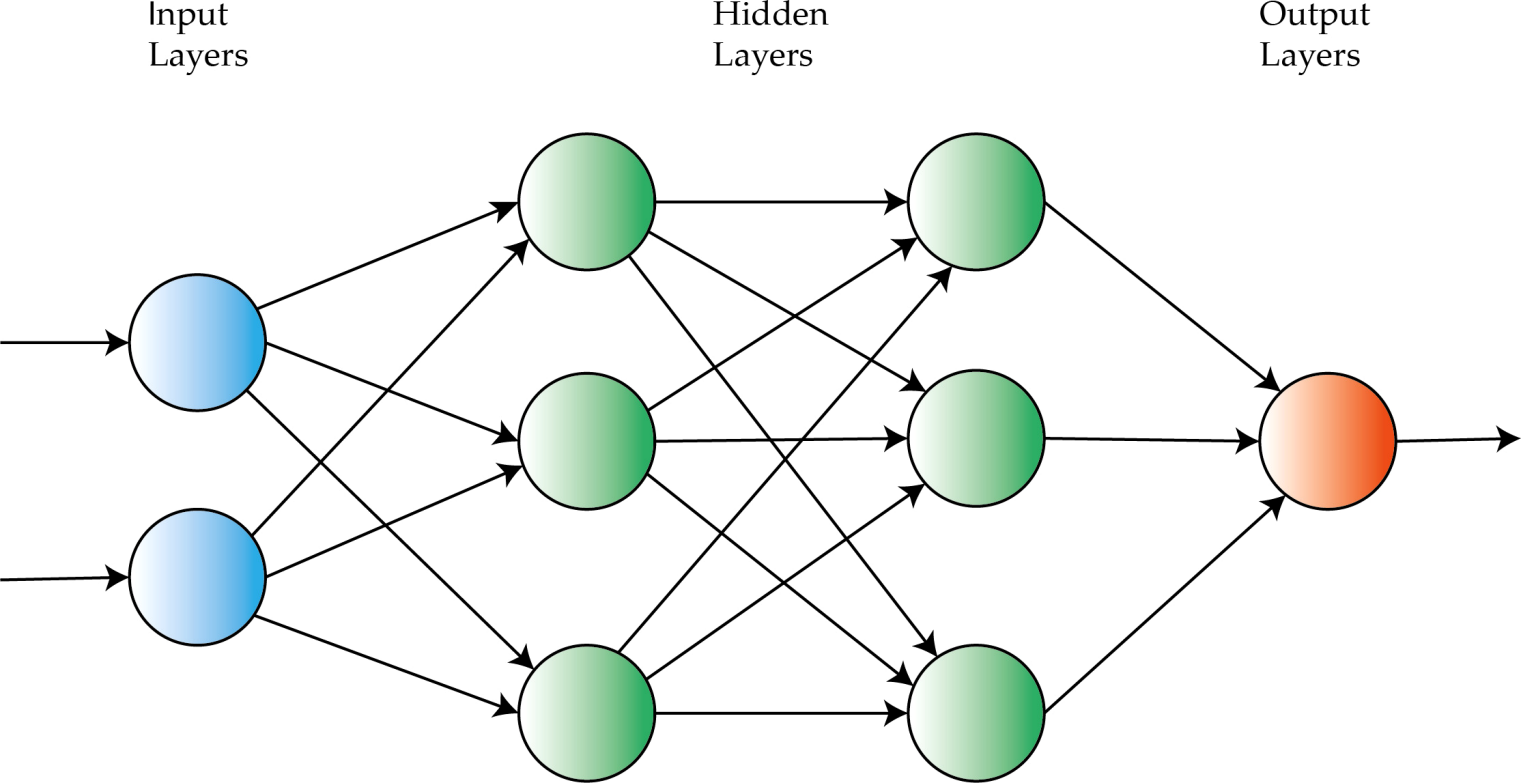
Visual representation of a neural network with two hidden layers.

Deep learning (DL) refers to techniques that rely on multiple layers of units (called neurons). Each neuron is a function that maps the input data to the desired output. In the training process, the network is capable of learning the parameters of such mappings. **Figure 14** shows the scheme of a network with two hidden layers. The name “deep” refers to the number of layers employed in these kinds of models (Goodfellow et al., 2016). The key feature of a deep learning model is its capability to make representations of unstructured data such as images or raw text (Osco et al., 2021).

Likewise, DL models are used in conjunction with RGB, multi-spectral, and hyper-spectral images, to perform different tasks concerning the assessment of forest health. Lin and Chuang (2021) used deep convolutional neural networks ResNet50, VGG19, and SegNet to extract features from aerial RGB pictures to perform tree classification. However the initial results showed poor performance based on accuracy; thus the authors proposed a simplification of the images using Principal Component Analysis, selecting only the most important features of the images. With this approach, SegNet reached an accuracy of 95%. The same task was performed by Onishi and Ise (2021), from aerial RGB images individual tree crowns were segmented, and each individual tree crown was used as the input data for the deep learning model, which was capable of categorizing seven different tree species and achieved an accuracy over 90%. Here the deep learning architectures were AlexNet, VGG16, Resnet18, and Resnet152, these were used for fine-tuning the model. A similar approach was done by Zhang C. et al. (2020), where a model using ResNet50 achieved an accuracy of 92.6%. In the work presented by (Feng et al., 2020), the authors investigated the results of using multi-temporal information in a recurrent convolutional neural network, for mapping vegetation using multiple-seasons aerial images. Hell et al. (2022) used PointCNN and 3DmFV-NET to perform the classification of coniferous, deciduous, and dead trees; from a LiDAR 3D cloud point, the results show that both networks are capable of differentiating between coniferous and dead trees, and it can reach an overall accuracy of more than 80%.

Pulido et al. (2020) used segmentation networks DetectNET, Faster R-CNN, and Single Shot Multibox Detector (SSD) to perform tree recognition from multi-spectral images in a forested area. The results show that, while traditional methods are capable of identifying trees, DL models outperform them and show improved metrics in areas where trees are clustered together. A similar task was performed by Hao Z. et al. (2022), herein the authors used Mask region-bases convolutional neural networks (Mask R-CNN) and evaluated the effect of reducing the number for training. The results show that by randomizing the training dataset, thus training the model with dissimilar samples each time, the metrics of the model are not as affected; therefore the training images can be reduced.

The creation of segmented images of fire-prone vegetation areas can be achieved with the use of deep learning techniques, Trencanová et al. (2022), trained U-NET network to identify these areas from RGB images, and the results show an F1 score of 0.7 in the validation dataset; however, due to the complex labeling process, the authors suggest that further improvements are needed to enhance this technique of identifying areas in landscapes.

Liu et al. (2021) proposed a 3D deep learning structure called LayerNet to perform tree classification tasks, the network used as input individual tree point clouds obtained from a LiDAR point cloud, the advantage of the network is that it can be trained from disorganized 3D point clouds. Compared to other algorithms such as random forest or KNN, this method achieved an accuracy of 88%, greatly outperforming the other two more common methods, which also need to pre-process the information to reduce the dimensions of the data, thus reducing potentially valuable traits.

Deep learning can be used to determine canopy cover in a densely forested area. Li et al. (2022) use a deep learning approach to distinguish background vegetation points from over-story canopy points, to produce canopy maps from forests’ 3D imagery.The results show that the deep learning approach outperforms traditional canopy mapping methods, therefore it is an accurate and robust method for creating canopy maps under different illuminations and terrain conditions.

Regression tasks can be performed using deep neural networks, Babaeian et al. (2021) used several machine learning methods and compared them to neural networks with two or three depth layers; the input data were multi-spectral VIs, and texture measurements from the soil and the expected output was soil moisture content; the results indicate an error below 5% and a high correlation value between the machine learning models and the predicted output.

### 4.9 Other algorithms

Other machine learning algorithms have been sparsely applied in different tasks. For example, gradient boosting machines (GBM) have been used to estimate soil moisture content in vegetated areas. Babaeian et al. (2021) tested several ML algorithms to predict soil moisture content including GBM. The results yielded that Neural Networks outperformed the other algorithms based on prediction error and the correlation factor. In the study presented by Villacrés and Cheein (2022), boosting gradient machines were used to reconstruct vegetation indices. Another task accomplished by GBM is the prediction of leaf nitrogen content based on hyperspectral indices, this is done by Raj et al. (2021), where the model achieved a correlation factor of 0.63, in areas with water-stressed vegetation; however, the model didn’t achieve the same results in well-irrigated areas.

A more optimized version of gradient boosting is Extreme Gradient Boosting machine (XGB), this approach was used by Yu et al. (2021), to determine the forest structure and it was compared to random forest and support vector machines algorithms, in this studyit was determined that XGB was the best algorithm for this task achieving an F1 score of 0.91.

For classification purposes, Yang and Kan (2020) studied the use of Extreme learning machine (ELM) which is based on neural networks; and a Linear Bayes Normal Classifier (LBNC); the authors compared both algorithms with KNN; in this study ELM and LBNC achieved an accuracy of 97.55% and 96.53% respectively, both outperforming KNN in tree classification task.

The generation of digital terrain models was explored with the aid of machine learning (Arevalo-Ramirez et al., 2022), using conditional random field (CRF) to extract ground points; this approach generated smoother terrain models than other approaches not based on machine learning methods.

## 5 Discussion

**Figure 15:**
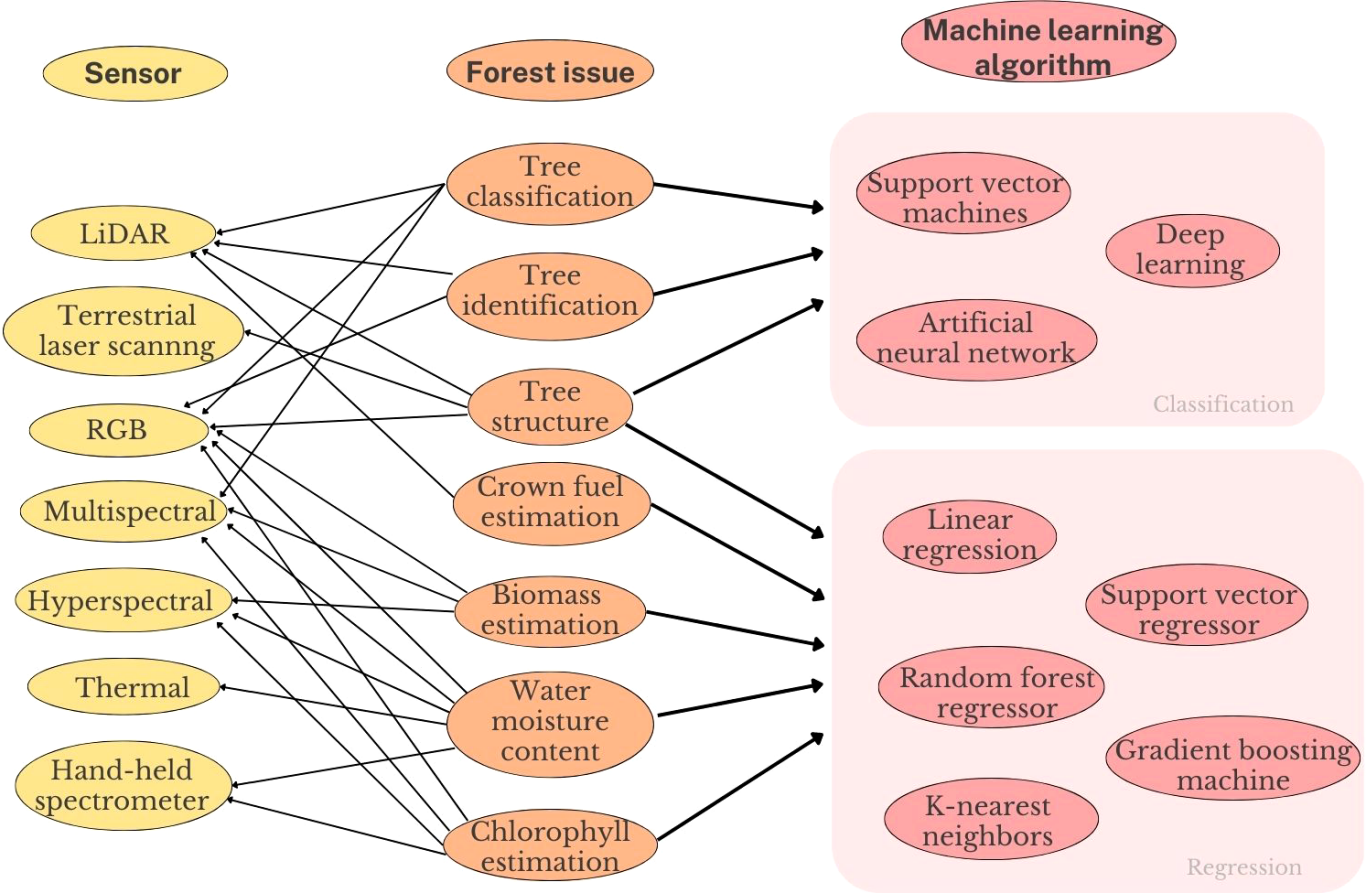
Relation between the vegetative or forest issue studied, the sensors and the machine learning algorithm chosen to do the investigation.

There is a clear relationship between the discussed vegetative or forest issues, the sensors, and the machine learning algorithms selected to accomplish the research objectives. For tasks such as tree recognition and classification, deep learning and other classification algorithms prevail, and the selected sensors for this task are mainly imaging systems, RGB, or multi-spectral. Other tasks corresponding to determining and predicting phenotype features of forests such as chlorophyll, water, and moisture content often use regression algorithms, where input data are the VIs gathered from RGB, multi-spectral, and hyperspectral cameras. In the case of physical modeling of forests and determining its parameters, sensors such as LiDAR or terrestrial laser scanning systems are more suitable, due to their capability of creating 3d models from point clouds. **Figure 15** illustrates the relationship between the vegetative issues, the sensors, and the data processing algorithms.

In general, all the reviewed works follow a somewhat similar workflow described by Müllerová et al. (2021): a problem in forestry health assessment is identified (chlorophyll prediction, water content estimation, biomass estimation, forest structure parametersestimation, tree classification, crown fuel estimation). Then the suitable sensors are selected depending on the needs of the problem, for example, if the problem is related to the geometric features of forests, a LiDAR sensor could fulfill the requirements. RGB, multi-spectral, and hyper-spectral cameras are more suited when spectral information is required and VIs are needed for example in chlorophyll estimation. The specific spectral response can also be used as an indicator of a specific tree speciesthus VIs are ideal to perform tree segmentation. Once the sensors are chosen, the data acquisition process is conducted. One of the most difficult parts of assessing forest health is the information processing phase. There is no clear pathway that leads to a correct decision when deciding which algorithm is the best to process the information according to the needs; as shown in the previous section, machine learning algorithms are a powerful alternative to process data and reach meaningful results.

### 5.1 Sensors used in remote sensing for forestry health assessment

Forestry health assessment aided by machine learning and remote sensing platforms is a promising trend in recent years. With the evolution of technology and machine learning techniques, better results in predictions of factors that affect forestry health have been accomplished. It is now possible to determine features from hyperspectral and multi-spectral imaging technologies, the use of UAVs helps the survey of great areas in short time, contrasted with a visual inspection from experts.

The use of LiDAR technology allows precise 3D reconstruction of environments in the range of centimeters (Hologa et al., 2021), allowing a complete geometrical characterization of forests, and the retrieval of tree and forest structure parameters. Efforts of mapping are important for forestry health assessment and to test algorithms; (Webster et al., 2018) performed thermal characterization of forest canopies in a large survey area, the study also made a coincident RGB mapping of the area, facilitating the access to public data to the scientific community.

The use of multi-spectral and hyper-spectral cameras to detect leaf reflectance and to compute different VIs has allowed an improvement in prediction techniques with the aid of machine learning algorithms. However, the information that can be gathered from spectral imaging methods is vast, and most of it will not have any correlation with the desired measurement, thus it is a current challenge to discover which bands and VIs are suitable for the different tasks in forestry health assessment. One way of reducing the dimensionality of input data for machine learning algorithms is the use of statistical methods to determine which information is more valuable and will provide better insight into the process, a common practice to reduce the dimensionality is to perform principal component analysis (PCA). Shovon et al. (2022) performed PCA in multi-spectral images, then a new VI with the four principal components, which was useful for identifying trees from snags. In the work presented by Kopacková-Strnadová et al. (2021), PCA was performed to reduce four spectral bands to three (three principal components), and with the selected bands, a VI was computed to predict photosynthetic pigments (i.e Chlorophyll). A similar process was performed by Barber et al. (2021), where the authors reduced the number of bands to predict fuel moisture in grasslands, again Ahmed et al. (2021b), reduced the number of multi-spectral bands to three principal components that represented the 86% variability of the images to generate VIs for tree identification. There is a greater issue when using hyperspectral imaging cameras since they can provide up to hundreds of bands; Yang and Kan (2020) retrieved 114 bands from a hyper-spectral camera, using a reduction process 14 bands were selected as principal feature bands, greatly reducing the dimension of the data.

### 5.2 Machine learning in forestry applications

The current trend in remote sensing for forestry health assessment is to use machine learning methods to process the information and find the desired correlations. These novel techniques currently outperform other methods that do not involve a training process, for example in the tree classification task Shovon et al. (2022) presented a thresholding algorithm to perform tree classification task, and even though the results were considered satisfactory, they are greatly outperformed by deep learning methods using convolutional layers. The accuracy is near a 90% (Onishi and Ise, 2021) on the training dataset with seven different tree species, whereas (Shovon et al., 2022) reported an accuracy of 80%.

The studies in classification tasks highlight that the use of deep learning techniques greatly outperforms other classification techniques (Onishi and Ise, 2021; Hell et al., 2022), and other studies present the advantage that the data does not need pre-processing (Liu et al., 2021). Hao Y. et al. (2022) performed individual tree detection without using machine learning models, and even though the proposed method improves the detection accuracy, reaching 90% in some scenarios; it is outperformed by the deep learning algorithm conducted proposed by Hao Z. et al. (2022).

The information needed as input data for deep learning and machine learning techniques is not clear either; in some cases, data extracted from UAV flights in a particular season of the year is insufficient for regression and classification purposes; thus recent articles investigate the use of multi-temporal data, for example, the results presented by Kopacková-Strnadová et al. (2021) suggest that temporal data is needed for predicting photosynthetic pigments in trees, given the fact that VIs from leaves of a certain age yielded the stronger correlated models. Other studies (Imangholiloo et al., 2020), explored the option of using data from different seasons for characterizing seedlings. Feng et al. (2020) used multi-temporal data to train DL networks, improving the accuracy of the model by more than 20% compared to the model using mono-temporal information.

For regression purposes, there is no clear tendency in the techniques that can be used to retrieve the desired data and make the predictions with the least amount of error. Most of the studies that rely on a prediction value, train different machine learning algorithms and assess the performance of each one using quantitative metrics. The performance of the algorithms varies case by case.

#### 5.2.1 Publicly available data

One of the biggest drawbacks of using machine learning is the lack of curated available data to train the algorithms. In most forestry health assessment applications, not only the data acquisition process is necessary, but also generating the ground truthis needed. Generally, the ground truth is acquired with the help of expert knowledge and *in situ* measurements, which is an expensive and time-consuming process; thus studies to create large datasets fulfill a vital role for the scientific community. Weinstein et al. (2021) created a dataset containing LiDAR, RGB, and hyper-spectral information, with manual delineation of individual tree crowns. This dataset can be used to train machine-learning algorithms for tree detection and classification. Other studies compared how the reduction of samples affects the performance of deep learning models. Hao Z. et al. (2022) showed that by randomizing the training dataset and creating more dissimilar samples it is possible to reduce the number of training images without affecting the performance of the model. Research about the retrieval of pigments, particularly chlorophyll, water, and moisture content, is conducted through spectral information at the leaf or canopy level. Several datasets containing samples of multiple leaves and their reflectance are of great help when developing machine learning models for regression purposes, using as input some form of spectral data. Among the most used datasets for these purposes are the following: ANGERS (Jacquemound et al., 2003), which contains the spectral reflectance of 276 live, fresh leaves of 39 species of trees located in Angers, France; alongside chemical and physical measurements such as chlorophyll content and water content. Another dataset of similar characteristics is LOPEX dataset (Hosgood et al., 1993), which presents reflectance data of 330 leaf samples from 45 different tree species, this dataset also presents biochemical properties for the dataset. Both datasets and other similar ones can be found online (https://ecosis.org/). One important model for remote sensing applied to forestry applications is the PROSPECT model (Feret et al., 2008), which recreates spectral reflectance and transmittance at the canopy level, and could be of great use when predicting biochemical properties of leaves including pigment content (Feret et al., 2008). Information about publicly available datasets, including ANGERS, LOPEX and the one presented by Weinstein et al. (2021) is summarized in **Table 3**

**Table 3:** Publicly available datasets for forestry health assessment.

Dataset	Content	Information	Case Application
ANGERS	Information from 276 leaves of different species	Visible and infrared spectra. Physical measurements. Biochemical analysis (Pigment content)	Development of model PROSPECT5 for reconstructing leaf reflectance (Feret et al., 2008). Testing machine learning algorithms for pigment estimation (Koirala et al., 2020; Shi et al., 2022).
LOPEX	Information from 330 samples of different species	Visible and infrared spectral. Physical Measurements. Biochemical Analysis (Pigment content).	Development of model PROSPECT5 (Feret et al., 2008). Training machine learning algorithms for pigment estimation Koirala et al. (2020)
Dataset presented by Weinstein et al., 2021).	Multiple sensor data and individual crown delineation.	RGB images. Hyper-spectral images. LiDAR point cloud. Individual image-annotated crowns. Individual field annotated crowns.	Development of individual crown detection algorithms from RGB and hyper-spectral images, and LiDAR point clouds Weinstein et al., 2021).

Datasets for forestry applications using deep learning are scarce and, in the reviewed works, every group of researchers created its own databases with annotations, for their intended objectives. However public information is available and it has been compiled at Diez et al. (2021).

#### 5.2.2 Big data approaches

Another future perspective for the assessment of forest health is the use of big-data approaches; under this new perspective, it is possible to use in conjunction with information retrieved from various sources including satellite platforms, airborne and terrestrial vehicles, and *in-situ* measurements to model the ever-changing dynamic of forests. One approach is to use the geological information-modeling system (GIMS), as presented by Varotsos and Krapivin (2017), who used GIMS to perform simulations evolution of the climate-nature-society system.

### 5.3 Future perspectives for machine learning and remote sensing in forestry health assessment

As shown in this current work, remote sensing aided by machine learning algorithms for forestry health applications is an active research field. As the methods of processing information advance and become more sophisticated, there is the possibility of highly improved forest management practices and contributing to sustainable forest management. Various studies (Liu et al., 2021; Onishi and Ise, 2021; Hell et al., 2022; Shovon et al., 2022), reported improved results in the metrics for tree recognition and tree classification, demonstrating the capabilities of machine learning to generate more precise models.

Another area that will continue to benefit from the improvement of models is the area of wildfire prevention (Jain et al., 2020). Correctly predicting fuel moisture content and biomass is of great help for predicting areas prone to wildfires. As seen in the reviewed works (Cężkowski et al., 2020; Raddi et al., 2021; Wells et al., 2021; Yao et al., 2021; Narmilan et al., 2022; Nasiri et al., 2022; Torre-Tojal et al., 2022), ˙ the use of machine learning algorithms have helped researchers predict biomass of fine fuels and moisture content at leaf and canopy level; thus helping identify dangerous areas for wildfire prevention. Machine learning models, alongside remote surveillance, carried out by UAV or satellite platforms will be of great importance for the prevention of disasters and the correct decision-making in disaster areas Jain et al. (2020).

## 6 Conclusions

The current state of the art suggests that for regression purposes (i.e estimating tree features, chlorophyll content, water leaf content, and soil moisture content among others); machine learning techniques are suitable. Choosing the imaging systems or sensors depends on the appropriate input data for the model it could be in the form of multi-spectral indices or metrics derived from LiDAR point clouds. However, there is no consensus on which regression technique achieves better performance.

DL techniques are a common trend for tree identification and classification tasks; these methods outperform other classification algorithms such as SVM and random forests, but they present the withdrawal of not enough data for training and validation purposes.

Most recent research is using multi-temporal information to improve the classification of trees from aerial images since the growing stage of trees affects their physical and chemical features.

The characterization of forests and their structure is a complex task due to the nature of the terrain, mixed and dense vegetation, constant evolution due to natural causes (different growth stages of the trees), and external causes (droughts, wildfires, climate change); therefore similar methodologies might not be suitable depending on the ecosystem.

The reviewed articles suggest that assessing forest features through remote sensing and machine learning techniques is a viable trend; since many ML techniques are being used for predicting forest health indices. Most recent works started exploring the use of Deep Learning Models, particularly convolutional neural networks to perform tree classification and recognition; these algorithms show great promise in reducing time for forest inventory and management, however; generating data for the training process, and creating models for general purposes are still some barriers in the use of deep learning techniques.

## Author contributions

All authors listed have made a substantial, direct, and intellectual contribution to the work and approved it for publication.
